# Triiron Complex with *N*-Ferrocenyl Aminocarbyne Ligand Bridging a Diiron Core: DFT, Electrochemical, and Biological Insights

**DOI:** 10.1021/acs.inorgchem.3c03408

**Published:** 2024-01-03

**Authors:** Chiara Saviozzi, Lorenzo Biancalana, Tiziana Funaioli, Marco Bortoluzzi, Michele De Franco, Massimo Guelfi, Valentina Gandin, Fabio Marchetti

**Affiliations:** † Department of Chemistry and Industrial Chemistry, University of Pisa, Via G. Moruzzi 13, I-56124 Pisa, Italy; ‡ Department of Molecular Science and Nanosystems, University of Venezia “Ca’ Foscari”, Via Torino 155, I-30170 Mestre (VE), Italy; § Department of Pharmaceutical and Pharmacological Sciences, University of Padova, Via F. Marzolo 5, I-35131 Padova, Italy

## Abstract

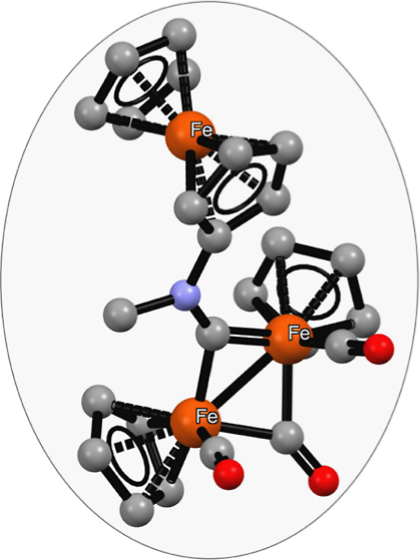

The first *N*-ferrocenyl aminocarbyne complex, [Fe_2_Cp_2_(CO)_2_(μ-CO){μ-CN(Me)(Fc)}]CF_3_SO_3_ (**[2]CF**_**3**_**SO**_**3**_), was synthesized with an 88% yield from [Fe_2_Cp_2_(CO)_4_], isocyanoferrocene (CNFc), and methyl triflate. The synthesis proceeded through the intermediate formation of [Fe_2_Cp_2_(CO)_3_(CNFc)], **1**. Multinuclear NMR experiments revealed the presence of cis and trans isomers for **[2]CF**_**3**_**SO**_**3**_ in organic solvents, in agreement with DFT outcomes. Electrochemical and spectroelectrochemical studies demonstrated one reduction process occurring prevalently at the diiron core and one oxidation involving the ferrocenyl substituent. The oxidation process is expected to favor the redox activation of **[2]**^+^ in a biological environment. Both **[2]CF**_**3**_**SO**_**3**_ and its phenyl analogue [Fe_2_Cp_2_(CO)_2_(μ-CO){μ-CN(Me)(Ph)}]CF_3_SO_3_ (**[3]CF**_**3**_**SO**_**3**_), prepared for comparison, exerted moderate antiproliferative activity against the human cancer cell lines A431, HCT-15, PSN-1, 2008, and U1285. However, **[2]CF**_**3**_**SO**_**3**_ exhibited a higher cytotoxicity than **[3]CF**_**3**_**SO**_**3**_, showed a substantial ability to induce intracellular ROS production, and outperformed cisplatin in a three-dimensional SCLC cell model.

## Abstract 2

The first *N*-ferrocenyl aminocarbyne complex, [Fe_2_Cp_2_(CO)_2_(μ-CO){μ-CN(Me)(Fc)}]CF_3_SO_3_ (**[2]CF**_**3**_**SO**_**3**_), was synthesized with an 88% yield from [Fe_2_Cp_2_(CO)_4_], isocyanoferrocene (CNFc), and methyl triflate.

## 1 Introduction

**Figure 1:**
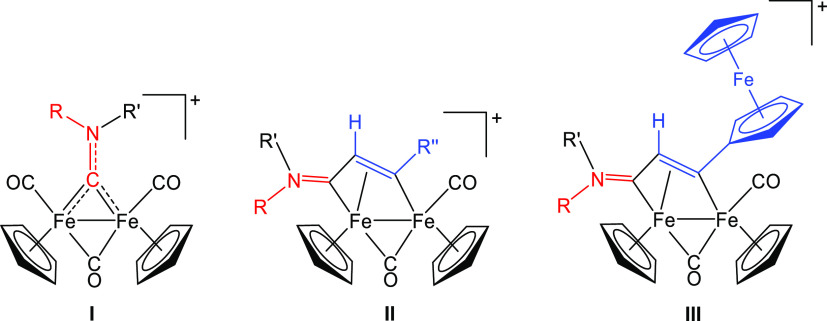
General structures of diiron aminocarbyne complexes (**I**) derived from [Fe_2_Cp_2_(CO)_4_], an isocyanide (CNR, red), and an alkylating agent (R′); their vinyliminium derivatives (**II**) resulting from the incorporation of an alkyne (HC≡CR″, blue); and including the case of HC≡CFc (**III**). CF_3_SO_3_^–^ or BF_4_^–^ salts. R, R′ = alkyl/aryl groups; R″ = H, alkyl, aryl, pyridyl, thiophenyl, carboxylate, and SiMe_3_.

The ferrocene skeleton (FeCp_2_, Cp = η^5^-C_5_H_5_) possesses unique redox behavior, low toxicity, and outstanding stability, and these properties have stimulated research in diverse fields.^1−3^ In particular, the ferrocene scaffold has garnered significant attention for formulating new anticancer metallodrugs.^4,5^ Ferrocenyl compounds typically exert their antiproliferative activity by undergoing Fe^II^ to Fe^III^ oxidation in the tumor environment; this electron transfer disrupts cellular redox homeostasis, ultimately leading to cell death.^6,7^ To harness the beneficial characteristics of ferrocene and create synergistic effects, synthetic chemists have pursued conjugation strategies. Basically, these strategies involve modifying one or two cyclopentadienyl rings of FeCp_2_ with suitable functionalities (e.g., phosphine groups, tetradentate Schiff bases, and NHCs) capable of binding to another metal center.^8,9^ Concerning potential medicinal applications, various metal structures with documented biological activities have been attached to ferrocene. Examples include ruthenium(III) complexes analogous to NAMI-A,^10^ platinum(II) complexes,^11,12^ and Ru(II)-arene complexes (Figure 1).^13−15^

Expanding the nuclearity of iron complexes by introducing the ferrocenyl unit can potentially enhance the properties and robustness of the resulting polyiron structures when compared to their lower nuclearity counterparts.^16,17^ In particular, having iron centers in distinct oxidation states may open up multiple redox pathways. For example, attaching a phosphino-ferrocene ligand to a diiron carbonyl core, serving as a model for the active site of [FeFe]-hydrogenases, was previously found to optimize the redox catalytic performance of the resulting mixed-valence triiron complex.^18^ Interestingly, despite its potential, this approach has been under-explored in the field of medicinal chemistry.

The commercially available compound [Fe_2_Cp_2_(CO)_4_] offers an excellent platform for investigating novel reactivity patterns due to its cost-effectiveness and the cooperative effects that arise from the two adjacent metal centers.^19−21^ Two primary classes of derivatives of [Fe_2_Cp_2_(CO)_4_], featuring a bridging cationic ligand, can be readily obtained through the sequential assembly of an isocyanide, an alkyl cation, and an alkyne (Figure 1, structures **I** and **II**).^22^ These two families of diiron compounds possess a rare combination of desirable prerequisites for medicinal applications, including a straightforward synthetic procedure, extended structural variability, adequate water solubility enhanced by their ionic nature, and substantial stability in aqueous media.^23^ We recently unveiled the promising anticancer potential of selected compounds of types **I**(24) and **II**.^25−27^ Additionally, we discovered that the incorporation of the ferrocenyl moiety within **II** using alkynyl ferrocene, CpFe(η^5^-C_5_H_4_C≡CH), enhances the antiproliferative activity of the resulting complexes (Figure 1, structure **III**) compared to ferrocenyl lacking analogues.^28^

Building on this premise, we became interested in incorporating the ferrocenyl unit as a substituent of the compact amino-alkylidyne (aminocarbyne^29^) group in **I**. It is noteworthy that, while complexes **I** are normally indefinitely air-stable and robust in a wide range of solvents (including aqueous media), their stability might be sensibly reduced with certain nitrogen substituents (R, R′) bearing unfavorable electronic properties (e.g., acetyl/benzoyl groups) or steric bulkiness (*tert*-butyl).^22,30,31^

Adding a suitable electrophile to a pre-existing isocyanide ligand (M-CNR) represents the most common literature strategy for obtaining aminocarbyne ligands (M-CNRR′).^22,32^ Accordingly, the typical synthesis of **I** involves the initial reaction of [Fe_2_Cp_2_(CO)_4_] with an isocyanide, followed by N-alkylation.^33^

Isocyanoferrocene (CNFc) is the simplest isocyanide-decorated ferrocene, and its utilization in coordination chemistry has been relatively limited, possibly due to its commercial unavailability and the challenging and tedious synthesis.^34^ Specifically, examples of CNFc coordination to metal centers has been substantially limited to ruthenium,^35^ iron phthalocyanine,^36^ diiron thiolate,^37^ and chromium complexes, including the unique case of a homoleptic CNFc complex, i.e., [Cr(CNFc)_6_].^38^ The scarce development of this topic is underscored by the observation that chemical modification of the CNFc ligand has been mostly confined to gold complexes, where amine addition to coordinated CNFc resulted in its conversion into diamino–alkylidene.^39^

The scarcity of information in this context sharply contrasts with the extensive use of isocyanides as universal and versatile ligands in coordination chemistry.^40,41^

In this work, we introduce CNFc (for which an optimized synthesis is provided) as a ligand to the {Fe_2_Cp_2_(CO)_3_} framework and its unprecedented transformation into an aminocarbyne ligand. The overall assembly can be viewed as a triiron system consisting of a Fe^II^ center (ferrocenyl) and a Fe^I^–Fe^I^ dinuclear core. We present the structural and electrochemical characterization of the resulting triiron complex, along with a preliminary evaluation of the anticancer potential in both 2D and 3D cell models.

## 2 Results and Discussion

### 2.1 Synthesis, Spectroscopic Characterization, and DFT Analysis

**Scheme 1:**
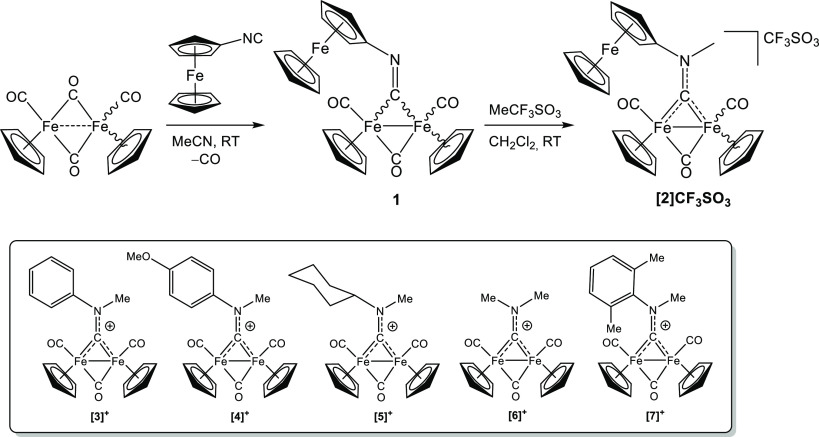
Two-step Synthesis of *N*-Ferrocenyl Aminocarbyne Diiron Complex **[2]CF**_**3**_**SO**_**3**_ via Intermediate Formation of an CNFc Adduct (**1**) The presence of isomeric forms is indicated with wavy bonds; the structures of the isomers of **1** are shown in Figure S3. Inset: related diiron aminocarbyne complexes (triflate salts) employed and/or discussed in this work; [**3**]^+^ is reported here for the first time, while [**4**]^+^,^24^ [**5**]^+^,^24^ [**6**],^+^^47^ and [**7**]^+^^46^ were previously described.

Initially, CNFc, was synthesized from ferrocene via the intermediate formation of aminoferrocene using a multistep procedure optimized with respect to the literature (for details, refer to the Experimental Section and the Supporting Information). Subsequently, the reaction between [Fe_2_Cp_2_(CO)_4_] and freshly prepared CNFc, in a 1:1 molar ratio, was conducted in acetonitrile at room temperature and proceeded with the selective substitution of one carbonyl ligand with CNFc (Scheme 1). This aspect is nontrivial, as it is documented that allowing [Fe_2_Cp_2_(CO)_4_] to react with one equivalent of isocyanides can lead to multiple CO–CNR substitutions, resulting in mixtures of products.^32^

Based on infrared spectroscopy measurements, [Fe_2_Cp_2_(CO)_3_(CNFc)], **1** - an uncommon example of an CNFc coordination complex (see Introduction) - is believed to exist in solution as a mixture of four interconverting isomers. These isomers vary in the relative orientation of the Cp ligands (cis or trans) and the coordination site of the CNFc ligand (terminal or bridging), see Figure S3. This situation aligns with the general case of [Fe_2_Cp_2_(CO)_3_(CNR)] complexes (R = alkyl or aryl).^32,42,43^ Exception arising when bulkier R groups (e.g., 2,6-dimethylphenyl and cyclohexyl) are present, disfavoring the bridging CNR coordination.^32^ The interconversion of isomers in solution was previously elucidated to follow the Adams–Cotton mechanism, which entails the formation of bridge-opened intermediates (comprising only terminal ligands bound to the diiron core), where rotation around the Fe–Fe bond is permitted.^32,44^

In the case of **1**, cis and trans isomers display the IR bands accounting for the terminal CO ligands at 1988 and 1951 cm^–1^, respectively (CH_2_Cl_2_ solution). Prior research has demonstrated that the isomeric composition of [Fe_2_Cp_2_(CO)_3_(CNR)] complexes is usually solvent-dependent, with polar solvents favoring the more polar cis isomer over the trans isomer.^45^ In agreement with this observation, both cis-**1** and trans-**1** were detected in comparable amounts in CH_2_Cl_2_ (μ = 1.60 D) solution, while cis-**1** was prevalent in MeCN (μ = 3.92 D), Figure S4.

The coordination mode of the CNFc ligand in **1** can be deduced from the corresponding CN stretching vibration, appearing at 2100 and 1691 cm^–1^ for terminal (C≡N) and bridging (C=N) coordination, respectively.^32,46^ A comparative analysis of IR spectra suggests that the bridging-isocyanide isomers are slightly favored in acetonitrile solution compared to dichloromethane (Figures S3 and S4). The wavenumber associated with the terminal CNFc is lower than that of noncoordinated CNFc (2122 cm^–1^ in CH_2_Cl_2_ solution), indicating a significant occurrence of Fe → CNFc π-back-donation in **1**.^32,41^

The crude solid residue obtained from [Fe_2_Cp_2_(CO)_4_] and CNFc was dissolved in dichloromethane and treated with methyl triflate, yielding the *N*-ferrocenyl aminocarbyne complex **[2]CF**_**3**_**SO**_**3**_. Notably, **[2]CF**_**3**_**SO**_**3**_ could be purified through alumina chromatography without decomposition^30^ and was isolated as an air-stable red solid in 88% yield (Scheme 1).

The ^1^H NMR spectrum of **[2]CF**_**3**_**SO**_**3**_ in CDCl_3_ revealed the presence of two sets of signals in an approximately 5:1 ratio, which were assigned to cis and trans isomers, respectively, based on NOESY evidence (Figures S6, S7, and S9). It is noteworthy that cis–trans isomerism has been rarely observed in diiron compounds of the type [Fe_2_Cp_2_(CO)_2_(μ-CO){μ-CN(R)(R′)}]^+^, and specifically only for R = R′ = Me or Et.^48^

**Figure 2:**
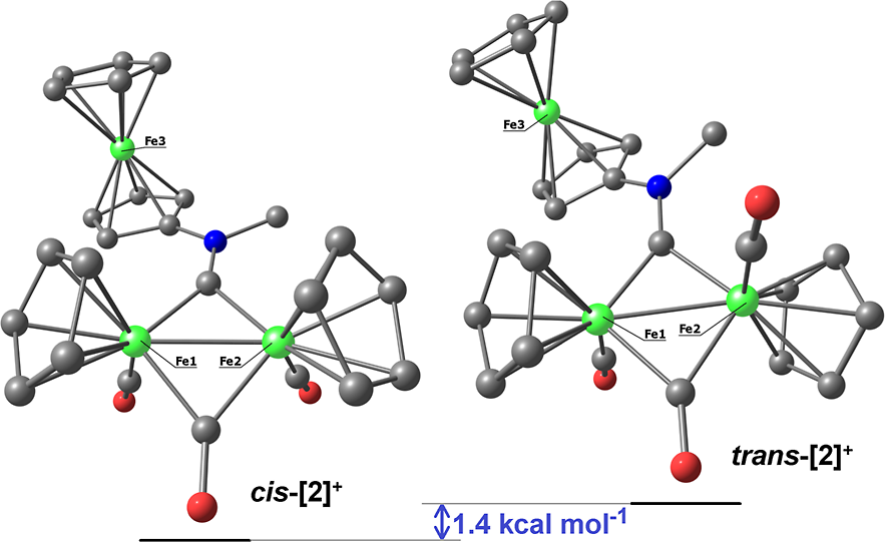
DFT-optimized geometries of cis-**[2]**^+^ (left) and trans-**[2]**^+^ (right), computed at PBEh-3c level (CHCl_3_ as a continuous medium). Fe, green; N, blue; and C, gray. Hydrogen atoms were omitted for clarity. Selected computed bond lengths for cis-**[2]**^+^ (Å): Fe1–C(carbyne) 1.856; Fe2–C(carbyne) 1.894; carbyne-*N* 1.297; Fe1–Fe2 2.482; Fe1–C(CO) 1.776; Fe2–C(CO) 1.774; Fe1–C(μ-CO) 1.935; Fe2–C(μ-CO) 1.893; average Fe1–C(Cp) 2.112; average Fe2–C(Cp) 2.115; and average Fe3–C(Cp) 2.058. Selected computed bond lengths for trans-**[2]**^+^ (Å): Fe1–C(carbyne) 1.841; Fe2–C(carbyne) 1.875; carbyne-N 1.298; Fe1–Fe2 2.505; Fe1–C(CO) 1.780; Fe2–C(CO) 1.780; Fe1–C(μ-CO) 1.933; Fe2–C(μ-CO) 1.905; average Fe1–C(Cp) 2.119; average Fe2–C(Cp) 2.116; and average Fe3–C(Cp) 2.056.

DFT calculations conducted on **[2]**^+^ (PBEh-3c method, CHCl_3_ as a continuous medium) pointed out that the cis isomer is more stable than the trans one by 1.4 kcal/mol. This energy difference corresponds to a predicted cis/trans ratio of ≈11, at 298 K. The DFT-optimized geometries of cis-**[2]**^+^ and trans-**[2]**^+^ are shown in Figure 2, and the most salient calculated bonding parameters are provided in the caption.

**Scheme 2:**
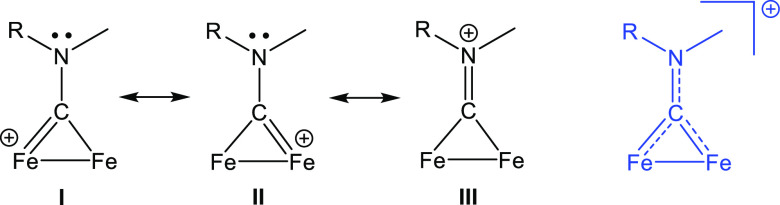
Bonding of the Bridging {CN(Me)R} Ligand (Including R = Fc as in **[2]**^+^) to the {Fe_2_Cp_2_(CO)_3_} Fragment: **I**, **II**: Aminocarbyne Resonance Structures; **III**: Iminium Resonance Structure In blue, comprehensive representation with a delocalized positive charge.

The geometric parameters extracted from the calculated DFT structures are in line with those observed in other diiron aminocarbyne complexes, as reported previously.^24,46^ In particular, the carbyne–nitrogen bond distance (1.297 Å in the cis isomer) is indicative of a partial iminium character. Besides, the average Fe-carbyne distance (1.875 Å in the cis isomer) is considerably shorter than the average Fe–μ–CO distance (1.914 Å in the cis isomer). These data highlight the greater π-acceptor ability of the (ferrocenyl)aminocarbyne ligand compared to the CO ligand.^22,49^ This leads the aminocarbyne to preferentially occupy a bridging coordination site over a terminal one, as such an arrangement enhances the electron backdonation from the iron atoms.^22^ The bonding situation in **[2]**^+^ can be therefore described in terms of the resonance forms depicted in Scheme 2. In detail, the competition for the vacant p-orbital on the carbyne carbon, between the nitrogen lone pair and the backdonation from the irons results in a substantial charge delocalization.

The experimentally detected cis/trans ratio (=5 in CDCl_3_ solution) is significantly lower than the theoretical value (11, in CHCl_3_ as a continuous medium). In general, the cis/trans isomerization for cationic tris-carbonyl μ-aminocarbyne complexes is not accessible at least up to 50 °C.^47,50^ Therefore, to promote a potential isomerization process, solutions of **[2]CF**_**3**_**SO**_**3**_ in various solvents were heated (Table 5). The greatest change in the cis/trans ratio was achieved by refluxing an acetone solution of **[2]CF**_**3**_**SO**_**3**_ for 2.5 h, resulting in an 18:1 cis/trans ratio. Consistently, DFT calculations considering acetone as an implicit solvent pointed out that cis-**[2]**^+^ is more stable than trans-**[2]**^+^ by approximately 1.7 kcal mol^–1^, corresponding to a cis/trans ratio of 17 at 298 K. However, some degradation was observed in all tested conditions, leading to the formation of non carbonyl byproducts that could not be identified. We presume that the isomerization process, if any, follows the Adams–Cotton mechanism via a terminal aminocarbyne species (see above).^22^ In the NMR spectra of **[2]CF**_**3**_**SO**_**3**_ (Figures S6–S8), each isomer displays nonequivalent Cp ligands within the {Fe_2_Cp_2_(CO)_3_} core due to the double bond character of the μ–C–N bond (vide infra), which hinders rotation around the C–N axis [e.g., for cis-**[2]**^+^: δ(^1^H) = 5.36, 4.84 ppm; δ(^13^C) = 90.5, 90.4 ppm]. The Cp ring belonging to the ferrocenyl moiety exhibits lower chemical shift values [δ = 4.43 (^1^H) and 70.5 ppm (^13^C) for cis-**[2]**^+^]. The predominant cis isomer displays ^13^C NMR resonances for the carbonyl ligands at 255.3 (bridging CO) and 209.1 and 207.9 ppm (terminal CO). Accordingly, the IR spectrum of **[2]CF**_**3**_**SO**_**3**_ (in CH_2_Cl_2_) contains three bands associated with the stretching vibrations of the terminal (2024 and 1992 cm^–1^) and bridging carbonyl ligands (1837 cm^–1^). Notably, the IR absorptions for the two isomers are almost identical, with the bands substantially overlapping (Figure S5).

**Table 1:** Formal Electrode Potentials (V, vs FeCp_2_ and, in Brackets, vs Ag/AgCl) and Peak-To-Peak Separations (mV) for the Redox Processes Exhibited in CH_2_Cl_2_ or THF Solutions and Aqueous Media by [**2**]^+^, [**3**]^+^, [**4**]^+^, and [**5**]^+^

	oxidation processes				reduction process	
compound	*E*_1_°	Δ*E*_p_^a^	*E*_2_°	Δ*E*_p_^a^	*E*_3_°	Δ*E*_p_^a^
[**2**]CF_3_SO_3_^b^	+1.28^e^ (+1.73)		+0.28 (+0.73)	80	–1.44 (−0.99)	83
**[3]CF**_**3**_**SO**_**3**_^b^	+0.97 (+1.42)	101			–1.41 (−0.96)	88
[**4**]CF_3_SO_3_^b^	+0.97 (+1.42)	95			–1.40 (−0.95)	84
[**5**]CF_3_SO_3_^b^	+0.95 (+1.40)	120			–1.41 (−0.96)	84
**[2]CF**_**3**_**SO**_**3**_^c^	+0.24 (+0.81)	85			–1.42 (−0.85)	85
**[3]CF**_**3**_**SO**_**3**_^c^	+1.03^e^ (+1.60)				–1.40 (−0.83)	80
[**4**]CF_3_SO_3_^c^	+1.05^e^ (+1.62)				–1.42 (−0.85)	80
[**4**]CF_3_SO_3_^d^	+0.90^e^ (+1.10)				–1.17 (−0.97)	96
[**5**]CF_3_SO_3_^d^	+0.90^e^ (+1.10)				–1.18 (−0.98)	96

a Measured at 0.1 V/s.

b In CH_2_Cl_2_/[^*n*^Bu_4_N]PF_6_.

c In THF/[^*n*^Bu_4_N]PF_6_.

d In phosphate buffer.

e Peak potential value for irreversible processes.

**Table 2:** Behavior of Diiron Aminocarbyne Complexes in Aqueous Solutions (See Experimental Section for Details)^a^

	**[2]CF**_**3**_**SO**_**3**_	**[3]CF**_**3**_**SO**_**3**_
solubility/mol·L^–^^1^	<3 × 10^–^^4^^b^	1.6 × 10^–^^3^
Log *P*_ow_	0.55 ± 0.05	–0.38 ± 0.02
residual complex % in D_2_O/CD_3_OD	62%	68%
residual complex % in DMEM-d/CD_3_OD	65%	74%

a Solubility in D_2_O (^1^H NMR, Me_2_SO_2_ internal standard) and octanol/water partition coefficient (Log *P*_ow_; UV–vis) at 21 ± 1 °C. Relative stability in D_2_O/CD_3_OD and DMEM-*d*/CD_3_OD mixtures (5:2 v/v) at 37 °C after 48 or 24 h, respectively (^1^H NMR, Me_2_SO_2_ internal standard).

b Below the lowest value of quantitation.

**Table 3:** Cytotoxic Activity of Diiron Complexes and Cisplatin in 2D Models

IC_50_ (μM) ± SD.	**A431**	**HCT-15**	**PSN-1**	**2008**	**U1285**
**[2]CF**_**3**_**SO**_**3**_	60 ± 14	33 ± 12	23.1 ± 4.6	32.9 ± 2.8	16.0 ± 1.3
**[3]CF**_**3**_**SO**_**3**_	75.1 ± 8.9	86.2 ± 0.5	>100	86 ± 11	50.8 ± 6.7
cisplatin	1.7 ± 0.3	13.9 ± 1.6	12.1 ± 2.8	2.1 ± 1.1	6.9 ± 1.1

Key spectroscopic features are those related to the {μ-CN} unit, manifesting in an infrared absorption at 1527 cm^–1^ and a ^13^C NMR resonance at 326.8 ppm. Literature data concerning diiron aminocarbyne complexes of general formula [Fe_2_Cp_2_(CO)_2_(μ-CO){μ-CN(Me)(R)}]^+^ range from 1522 (R = 2,6-C_6_H_3_MeCl) to 1604 cm^–1^ (R = Me) and 331.5 (R = 2,6-C_6_H_3_MeCl) to 315.5 ppm (R = Me), respectively, and clearly correlate with the electron donor properties of the R substituent.^22,24^ The values obtained for **[2]CF**_**3**_**SO**_**3**_ (R = Fc) approximate those of the analogous complex with R = 2,6-C_6_H_3_MeCl, outlining that the ferrocenyl moiety behaves electronically as an aryl within the cationic aminocarbyne moiety. We note that ferrocenyl has been previously regarded as an electron donor group in various systems; for instance, its donor capability has been estimated to exceed that of the methyl group in organophosphorus compounds,^51^ and to approximate that of the 4-aminophenyl within W propargylidyne complexes.^52^

For sake of comparison, we synthesized the unprecedented *N*-phenyl aminocarbyne complex [Fe_2_Cp_2_(CO)_2_(μ-CO){μ-CN(Me)(Ph)}]CF_3_SO_3_, **[3]CF**_**3**_**SO**_**3**_, following the two-step method described for **[2]CF**_**3**_**SO**_**3**_ (Scheme 1). Briefly, freshly prepared phenyl isocyanide (from phenyl formamide, see the Experimental Section) was allowed to react with [Fe_2_Cp_2_(CO)_4_] in acetonitrile at room temperature. This reaction resulted in the formation of the known dark-violet compound [Fe_2_Cp_2_(CO)_2_(μ-CO)(μ-CNPh)],^53−56^ which was isolated through alumina chromatography. Subsequent methylation with CF_3_SO_3_Me in CH_2_Cl_2_ followed by chromatographic purification, afforded a red powder of the aminocarbyne complex **[3]CF**_**3**_**SO**_**3**_. The latter was isolated in ca. 41% yield, together with a minor amount of [Fe_2_Cp_2_(CO)(CNPh)(μ-CO){μ-CN(Me)(Ph)}]^+^ (unprecedented). The moderate yield and the formation of byproducts appear ascribable to the thermal instability and the enhanced reactivity of phenyl isocyanide promoted by metal coordination. The thermal or photolytic displacement of CO from [Fe_2_Cp_2_(CO)_4_] using PhNC was previously found to give [Fe_2_Cp_2_(CO)_2_(μ-CO)(μ-CNPh)] in 10–50% yields, in admixture with the disubstituted derivative [Fe_2_Cp_2_(CO)_2_(CNPh)_2_].^51,52^ A similar reaction involving [Ru_2_Cp_2_(CO)_4_] resulted in the complete decomposition of the isocyanide.^44^ Additionally, [Fe_2_Cp_2_(CO)_2_(μ-CO)(μ-CNPh)] was obtained in low to modest yields (5–46%) from other monoiron or diiron precursors.^53,54^

The novel *N*-phenyl aminocarbyne complex **[3]CF**_**3**_**SO**_**3**_ is air-stable and was characterized by IR and NMR spectroscopy (Figures S10–S14). The spectroscopic data for the {μ-CN} moiety well match the corresponding ones for **[2]CF**_**3**_**SO**_**3**_ (IR absorption at 1527 and 1531 cm^–1^ and ^13^C NMR resonance at 326.8 and 324.5 ppm, respectively, in **[2]CF**_**3**_**SO**_**3**_ and **[3]CF**_**3**_**SO**_**3**_).

**Figure 3:**
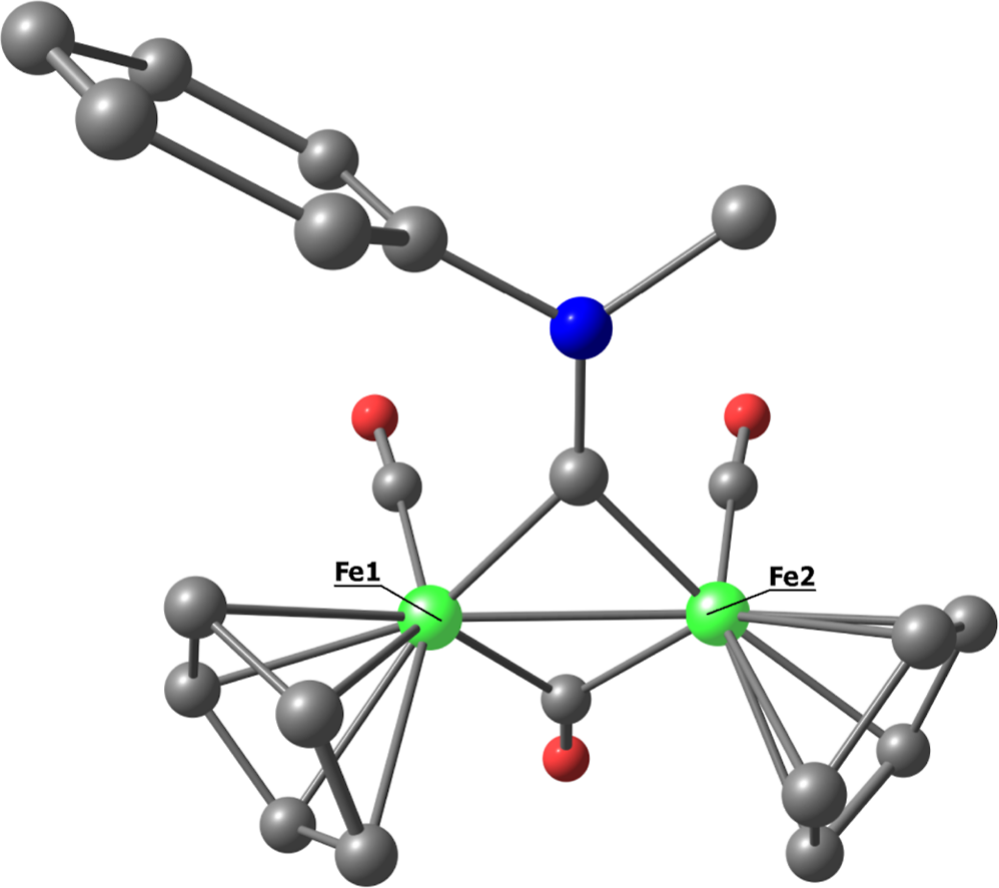
DFT optimized geometry of cis-**[3]**^+^, computed at PBEh-3c level (CHCl_3_ as a continuous medium). Fe, green; N, blue; and C, gray. Hydrogen atoms were omitted for clarity. Selected computed bond lengths: Fe1–C(carbyne) 1.859; Fe2–C(carbyne) 1.844; C(carbyne)-*N* 1.291; Fe1–Fe2 2.491; Fe1–C(CO) 1.774; Fe2–C(CO) 1.772; Fe1–C(μ-CO) 1.903; Fe2–C(μ-CO) 1.937; average Fe1–C(Cp) 2.105; and average Fe2–C(Cp) 2.107.

The cation **[3]**^+^ was computationally optimized at the C-PCM/PBEh-3c level, considering CHCl_3_ as the solvent. Interestingly, the cis isomer exhibited higher stability than the trans isomer by about 3.7 kcal mol^–1^. This outcome aligns with the experimental detection of a single species, cis-**[3]**^+^, in solution. The DFT-optimized structure of cis-**[3]**^+^ is shown in Figure 3 and selected computed bond lengths are collected in the caption. Figure S15 shows a comparative view of the cis and trans isomers of **[3]**^+^.

In order to compare the electronic properties of the ferrocenyl- and phenyl-substituted aminocarbyne ligands, extended charge decomposition analyses (ECDA)^57^ were carried out. A positive charge was assigned to the aminocarbyne moiety. The net electron transfer from {Fe_2_Cp_2_(CO)_2_(μ-CO)} to [CN(Me)Fc]^+^ is calculated to be 0.754, and a closely related value of 0.784 was determined for [CN(Me)Ph]^+^. This similarity of values suggests that, overall, the two ligands exhibit comparable coordination features and electronic trends, in agreement with the key spectroscopic data discussed above. Furthermore, it is remarkable that the carbonyl regions of the simulated IR spectra of **[2]**^+^ and **[3]**^+^ are superimposable, as evident in Figure S16.

### 2.2 Electrochemical Studies and DFT Results

The redox chemistry and the in situ IR spectroelectrochemistry (IR SEC) of **[2]CF**_**3**_**SO**_**3**_ and **[3]CF**_**3**_**SO**_**3**_ were investigated in dichloromethane and THF solutions containing [N^*n*^Bu_4_]PF_6_ (0.2 mol dm^–3^) as the supporting electrolyte. The aminocarbyne complexes **[4]CF**_**3**_**SO**_**3**_ and **[5]CF**_**3**_**SO**_**3**_ (Scheme 1) were included in this study for comparative purposes. Table 1 collects the formal electrode potentials of the observed redox changes.

**Figure 4:**
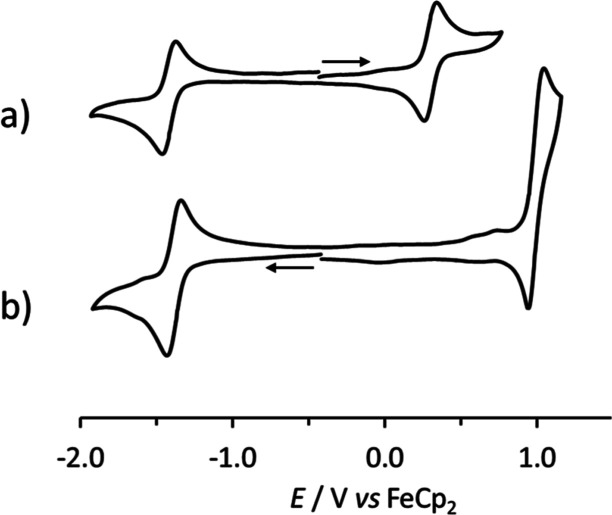
CV recorded at a Pt electrode in a CH_2_Cl_2_ solution of (a) [**2**]^+^ between −1.92 and +0.72 V; (b) [**3**]^+^ between −1.92 and +1.16 V. [^*n*^Bu_4_N]PF_6_ (0.2 mol dm^–3^) as a supporting electrolyte. Scan rate: 0.1 V s^–1^. Arrows indicate the direction of the scan.

The presence of the ferrocenyl substituent within [**2**]^+^ becomes evident from the comparison of the cyclic voltammetries of [**2**]^+^ and [**3**]^+^ in the positive potential region. In CH_2_Cl_2_ solution, [**2**]^+^ displays one diffusion controlled, monoelectronic oxidation at +0.28 V vs FeCp_2_. This process exhibits features of chemical reversibility in the cyclic voltammetric time scale (Figure 4a) and can be attributed to the Fe^II^-ferrocenyl moiety of the triiron complex. On the other hand, a two-electron, partially chemically reversible oxidation at a higher potential (about +0.97 V vs FeCp_2_) was observed in the CV profiles of **[3]**^+^ (Figure 4b), **[4]**^+^, and **[5]**^+^ (Table 1). This result seems consistent with a previous electrochemical study conducted on **[6]CF**_**3**_**SO**_**3**_ and **[7]CF**_**3**_**SO**_**3**_ in acetonitrile.^58^

**Figure 5:**
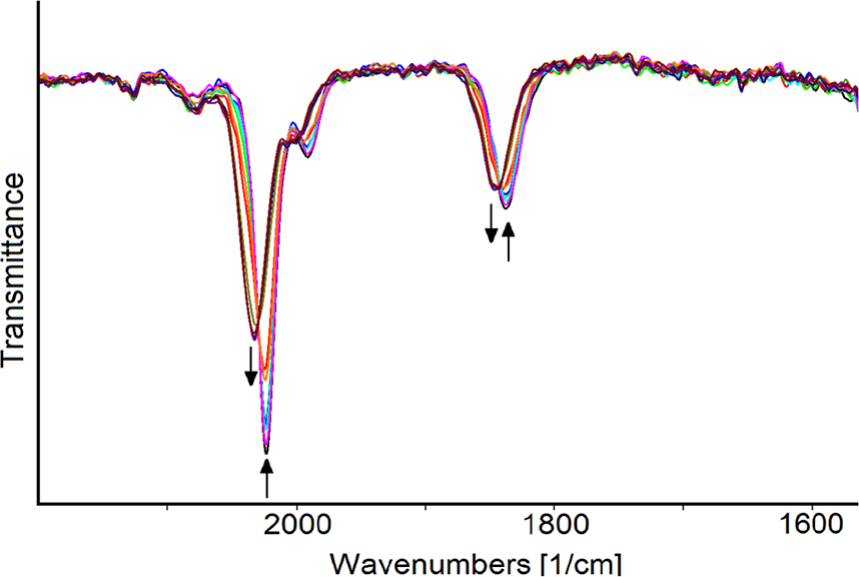
IR spectra of a CH_2_Cl_2_ solution of **[2]CF**_**3**_**SO**_**3**_ recorded in an OTTLE cell during the progressive increase of the WE potential from +0.1 to +0.5 V (vs FeCp_2_; scan rate 1 mV sec^–1^). [^*n*^Bu_4_N]PF_6_ (0.2 mol dm^–3^) as the supporting electrolyte. Absorptions of the solvent and supporting electrolyte have been subtracted.

When the oxidation process of [**2**]^+^ was investigated by in situ IR-SEC in an optically transparent thin-layer electrochemical (OTTLE) cell,^59^ a blueshift of the IR absorption bands due to CO-stretching modes of terminal and bridging CO ligands of [**2**]^+^ (from 2024, 1992, and 1837 cm^–1^ to 2033, 2002, and 1845 cm^–1^) was detected as the working electrode (WE) potential increased from +0.1 to +0.5 V (vs FeCp_2_), Figure 5. This shift corresponds to the quantitative formation of the one-electron oxidation product [**2**]^2+^. The latter remained stable within the time scale of the spectroelectrochemical experiment; indeed, the initial spectrum was almost completely restored during the reverse reduction scan.

The slight shift (approximately +10 cm^–1^) of the wavenumbers agrees with the assumption that the reversible one-electron removal primarily affects the ferrocenyl portion of the complex, thereby having minimal impact on the carbonyl ligands bound to the Fe_2_Cp_2_ core.

In the negative potential range, all the complexes [**2**]^+^–[**5**]^+^ undergo a monoelectronic, diffusion controlled, and electrochemically reversible reduction at approximately the same potential. However, this reduction is complicated by subsequent chemical reactions (with *i*_b_/*i*_f_ ratios of 0.84 and 0.83 at a scan rate of 100 mV s^–1^ for [**2**]^+^ and [**3**]^+^, respectively).

Collectively, the in situ IR SEC analyses of [**2**]^+^–[**5**]^+^ in CH_2_Cl_2_/[^*n*^Bu_4_N]PF_6_ solutions indicate that electron addition mainly involves the [Fe^I^Fe^I^] core.

As the potential of the working electrode gradually decreased from −1.3 to −1.6 V (vs FeCp_2_), the IR absorptions of [**2**]^+^ were initially replaced by new bands at lower wavenumbers (ν_CO_ = 1958, 1919, and 1741 cm^–1^, Figure S17a), attributed to the neutral radical species [**2**]^•^. However, prior to the complete disappearance of [**2**]^+^, a slight blueshift of the IR absorption bands due to the stretching modes of terminal and bridging CO ligands of the newly formed [**2**]^•^ was detected (ν_CO_ = 1963, 1928, and 1773 cm^–1^, Figure S17b). This process was completed within the following 10 min during microelectrolysis at a constant potential of −1.8 V (Figure S17c). Instead, the spectral changes recorded in the OTTLE cell over 10 min after the complete reduction of [**2**]^+^, without an applied potential, suggest that a disproportionation reaction of [**2**]^•^ occurs subsequent to the electron transfer. This reaction results in the regeneration of [**2**]^+^ and the formation of an unidentified species, 2* (ν_CO_ = 1963, 1928, and 1773 cm^–1^, Figure S18). Evidence of a disproportionation reaction of [**2**]^•^ and the bulk electrolysis to determine the electron stoichiometry of the transformation [**2**]^+^ → 2* (Figure S19) is supplied in the Supporting Information.

**Figure 6:**
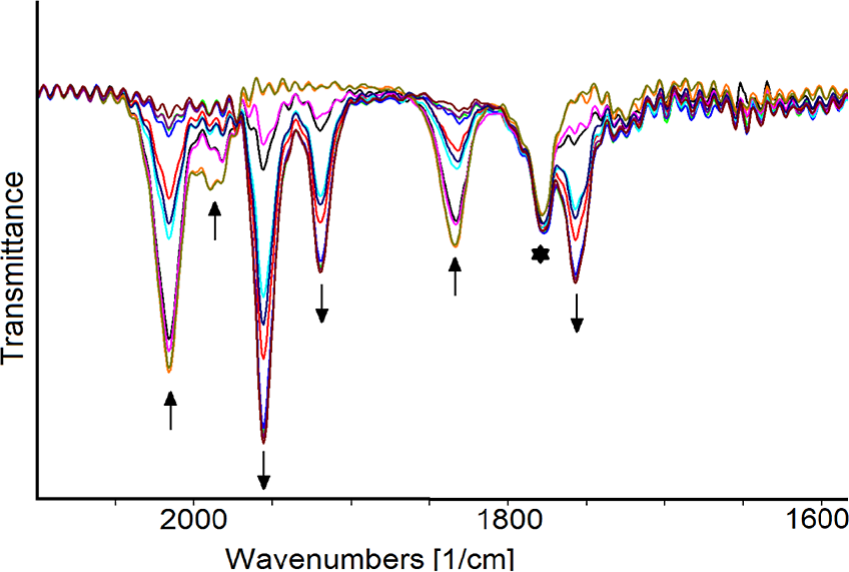
IR spectra of a THF solution of [**2**]^+^ recorded in an OTTLE cell during the progressive decrease of the WE potential from −1.2 to −1.6 V (vs FeCp_2_; scan rate 1 mV s^–1^) starred peak is due to impurities. [^*n*^Bu_4_N]PF_6_ (0.2 mol dm^–3^) as the supporting electrolyte. Absorptions of the solvent and supporting electrolyte have been subtracted.

The formation of the tris-carbonyl complex 2* (ν_CO_ = 1963, 1928, and 1773 cm^–1^) is restricted to the use of dichloromethane as solvent, and we hypothesize that it is consequent to the generation of radical species (X) from CH_2_Cl_2_, converting the bridging aminocarbyne ligand, CN(Me)(Fc), into a bridging aminocarbene, C(X)N(Me)(Fc).^46^ We verified that in THF/[^*n*^Bu_4_N]PF_6_ solutions, the reduction of [**2**]^+^ is fully reversible (*i*_b_/*i*_f_ = 1 at a scan rate of 100 mV s^–1^) (Table 1) and, consistently, an IR SEC experiment confirmed that the reduction of [**2**]^+^ (ν_CO_ = 2016, 1986, and 1834 cm^–1^) leads to the quantitative and entirely reversible formation of [**2**]^•^ (ν_CO_ = 1956, 1919, and 1757 cm^–1^), also in the long time scale of this experiment (Figure 6).

A parallel behavior was observed for [**3**]^+^–[**5**]^+^, in both CH_2_Cl_2_ and THF (Figure S20 refers to [**3**]^+^ as a representative compound), confirming that the reactivity of the radicals [**2**]^•^–[**5**]^•^ in dichloromethane is a general trend, which is related to the intervention of the solvent and independent of the presence of the ferrocenyl moiety.^60^ The large redshift of the IR absorption bands due to stretching modes of terminal and bridging CO ligands upon reduction (about 70 cm^–1^ for both complexes) suggests that the LUMO is mainly localized on the Cp_2_Fe^I^_2_ core, in agreement with previous DFT outcomes on the *N*-dimethyl derivative [**6**]^+^ (Scheme 1).^61^

**Figure 7:**
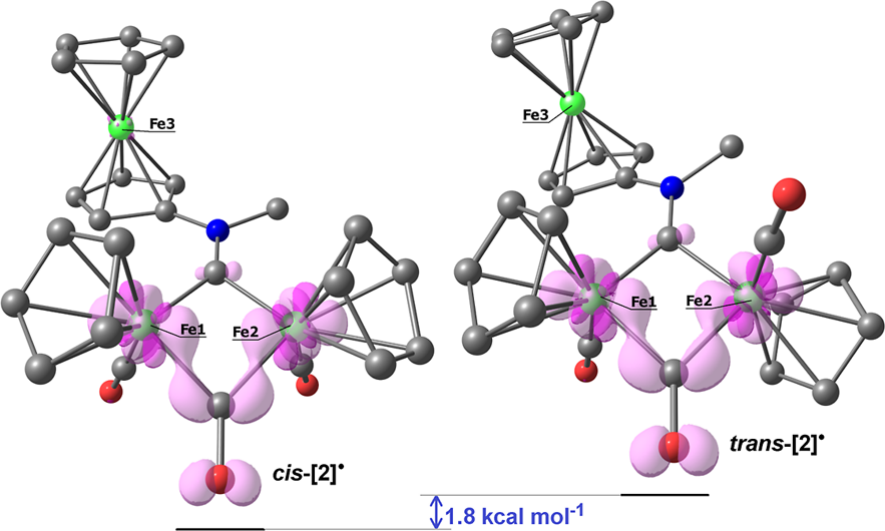
DFT-optimized geometries of cis-**[2]**^**•**^ (left) and trans-**[2]**^**•**^ (right), computed at PBEh-3c level (acetone as a continuous medium). Fe, green; N, blue; and C, gray. Hydrogen atoms were omitted for clarity. Spin density surfaces (isovalue = 0.01 au) in pink tones. Selected computed bond lengths for cis-**[2]**^**•**^ (Å): Fe1–C(carbyne) 1.886; Fe2–C(carbyne) 1.917; C–N 1.310; Fe1–Fe2 2.678; Fe1–C(CO) 1.743; Fe2–C(CO) 1.745; Fe1–C(μ-CO) 1.931; Fe2–C(μ-CO) 1.916; average Fe1–C(Cp) 2.176; average Fe2–C(Cp) 2.158; and average Fe3–C(Cp) 2.058. Selected computed bond lengths for trans-**[2]**^**•**^ (Å): Fe1–C(carbyne) 1.873; Fe2–C(carbyne) 1.899; C–N 1.311; Fe1–Fe2 2.714; Fe1–C(CO) 1.748; Fe2–C(CO) 1.752; Fe1–C(μ-CO) 1.930; Fe2–C(μ-CO) 1.930; average Fe1–C(Cp) 2.178; average Fe2–C(Cp) 2.158; and average Fe3–C(Cp) 2.057.

The structures of both the cis and trans isomers of the radical **[2]**^**•**^ were computationally optimized and are shown in Figure 7. The cis-**[2]**^**•**^ isomer resulted more stable than trans-**[2]**^**•**^ by approximately 1.8 kcal mol^–1^, considering acetone as the continuous medium. The optimized structures closely resemble those of the parent cations, exhibiting root-mean-square deviation (RMSD) values ranging from 0.118 to 0.133 Å. This outcome is in line with the electrochemical reversibility described above. The simulated IR spectra of cis-**[2]**^**•**^ and trans-**[2]**^**•**^, compared to the corresponding parent cationic complexes, highlight the significant shift of the carbonyl stretching vibrations toward lower wavenumbers (Figure S22). The spin density plots depicted in Figure 7 confirm that the reduction process involves the {Fe(μ-CO)Fe} fragment, while the contribution of the ferrocenyl unit appears negligible.

In summary, within the triiron species [**2**]^+^, the two iron-based redox systems (i.e., the ferrocenyl Fe^II^ and Fe^I^Fe^I^) are independent.

The redox properties of **[4]CF**_**3**_**SO**_**3**_ and **[5]CF**_**3**_**SO**_**3**_ were additionally investigated in a phosphate buffer (PB; pH = 7.3), given the sufficient solubility of these complexes in aqueous media. In the PB, the oxidation process for both [**4**]^+^ (Figure S21) and [**5**]^+^ is irreversible, multielectronic, and shifted at lower potentials by approximately 300 mV compared to the organic solvent.^62^ Conversely, a reduction occurs at potential values similar to those recognized in CH_2_Cl_2_ solution. In the backscan, we observed an absorption peak, probably due to the neutral complexes **4**^**•**^ and **5**^**•**^.

Anyway, both the reduction and oxidation potentials of [**4**]^+^ and [**5**]^+^ fall outside the biologically relevant potential range, which approximately covers the window −0.4 to +0.8 V vs SHE (−0.6 to +0.6 V vs Ag/AgCl).^63,64^ The limited water solubility of **[2]CF**_**3**_**SO**_**3**_ prevented its CV characterization in the PB solution. Nevertheless, if we assume that the behavior difference of **[2]**^+^ when varying the solvent parallels that of **[4]**^+^ and **[5]**^+^, the potential for achieving biooxidative activation could be accessible for the *N*-ferrocenyl aminocarbyne complex.

### 2.3 Behavior of Diiron Complexes in Aqueous Media and Biological Studies

In preparation for the biological investigation, we conducted an initial assessment of **[2]CF**_**3**_**SO**_**3**_ and **[3]CF**_**3**_**SO**_**3**_ in aqueous solutions. By using established ^1^H NMR and UV–vis methods, we assessed the water (D_2_O) solubility, the octanol–water partition coefficient, and the stability of the complexes under conditions relevant to biology (37 °C, cell culture medium), see Table 2. The change from a phenyl to a ferrocenyl group in the aminocarbyne ligand is accompanied by decreased water solubility and increased lipophilicity. Nevertheless, both **[2]CF**_**3**_**SO**_**3**_ and **[3]CF**_**3**_**SO**_**3**_ can be categorized as *amphiphilic* compounds (−0.5 < Log *P*_ow_ < +0.5). These compounds exhibited substantial stability at 37 °C, with 62–74% of the starting material remaining unchanged after 48 h in D_2_O solution or after 24 h in DMEM cell culture medium.^65^

Interestingly, ^1^H NMR spectroscopy showed for the aqueous solutions of **[2]**^+^ an enrichment in the trans isomer (cis/trans ratio ≈1), deviating from the situation observed in CDCl_3_ (see above). We did not collect evidence of an oxidation of the ferrocenyl unit in [**2**]^+^ in such aerobic environments (*E*° = +0.83 V vs SHE for O_2_/H_2_O at pH = 7).

Several diiron aminocarbyne complexes were previously investigated for their in vitro anticancer activity (see Introduction), which is strongly influenced by the nature of the aminocarbyne substituents (Figure 1, structure **I**). For instance, while **[6]CF**_**3**_**SO**_**3**_ does not exhibit cytotoxicity, **[4]CF**_**3**_**SO**_**3**_ and **[5]CF**_**3**_**SO**_**3**_ have showcased a significant antiproliferative activity against various human cancer cell lines.^23,24^ Their mechanism of action has been mainly associated with their capacity to interfere with cellular redox processes through the intracellular release of iron(I) species.^24^ The incorporation of the ferrocenyl in **[2]CF**_**3**_**SO**_**3**_ introduces an additional redox-active fragment that could potentially participate in intracellular biological processes (see Introduction). On the other hand, **[3]CF**_**3**_**SO**_**3**_, lacking the ferrocenyl unit, serves as a benchmark for comparison,^66^ especially when considering the similar electronic properties revealed by the bridging ligands CNMe(Fc) (in **[2]**^+^) and CNMe(Ph) (in **[3]**^+^), vide infra.

The cytotoxicity of **[2]CF**_**3**_**SO**_**3**_ and **[3]CF**_**3**_**SO**_**3**_ was evaluated across a range of human cancer cell lines representative of different solid tumors. In particular, the in-house cancer cell panel includes examples of human cervical (A431), colon (HCT-15), pancreatic (PSN-1), and ovarian (2008) carcinoma, as well as small-cell lung cancer (SCLC, U1285). The cytotoxicity parameters, expressed in terms of IC_50_ values acquired following 72 h of exposure to the MTT assay, are reported in Table 3. For comparison purposes, the cytotoxicity of the reference metal-based chemotherapeutic drug cisplatin was assessed under the same experimental conditions.

Cells [(3–8) × 10^3^ cell/well] were treated for 72 h with increasing concentrations of tested compounds. Cytotoxicity was assessed by MTT test. The IC_50_ values were calculated by the four-parameter logistic model (*p* < 0.05). SD is the standard deviation.

Both **[2]CF**_**3**_**SO**_**3**_ and **[3]CF**_**3**_**SO**_**3**_ showed a moderate cytotoxicity profile, with average IC_50_ values generally higher compared to cisplatin over the five tested human cancer cell lines. Within the diiron complexes, the ferrocenyl derivative **[2]CF**_**3**_**SO**_**3**_ is significantly more effective than the *N*-phenyl analogue **[3]CF**_**3**_**SO**_**3**_. On average, the in vitro antitumor activity of **[2]CF**_**3**_**SO**_**3**_ exceeded that of **[3]CF**_**3**_**SO**_**3**_ by a factor of 2.4. In particular, **[2]CF**_**3**_**SO**_**3**_ outperformed **[3]CF**_**3**_**SO**_**3**_ against pancreatic adenocarcinoma (PSN-1) and SCLC cells, showing 4-fold and 3-fold higher efficacy, respectively.

Although 2D cell cultures are widely employed for in vitro screening due to their low cost, simplicity, and reliability, they are unable to mimic the properties of in vivo solid tumors. In contrast, 3D cell cultures offer greater efficiency in reproducing the heterogeneity and complexity of tumor masses, and therefore they are more predictive of in vivo outcomes than traditional 2D cell cultures.^67^ On these bases, the cytotoxicity of **[2]CF**_**3**_**SO**_**3**_ and **[3]CF**_**3**_**SO**_**3**_ was also examined using a 3D cell culture model of SCLC cells. Hence, U1285 cells were treated with the investigated compounds for 72 h, and cell viability was assessed by means of the acid phosphatase (APH) assay (Table 4).

**Table 4:** Cytotoxic Activity of Diiron Complexes and Cisplatin in a SCLC 3D Model

IC_50_ (μM) ± SD.	U1285
**[2]CF**_**3**_**SO**_**3**_	51.3 ± 4.2
**[3]CF**_**3**_**SO**_**3**_	>100
cisplatin	65.4 ± 1.4

Spheroids (2.5 × 10^3^ cells/well) were treated for 72 h with increasing concentrations of tested compounds. The growth-inhibitory effect was evaluated by means of the APH test. IC_50_ values were calculated from the dose–survival curves using a four-parameter logistic model (*p* < 0.05). SD = standard deviation.

Remarkably, the 3D cytotoxicity studies not only confirmed the superior anticancer potential of the ferrocenyl derivative **[2]CF**_**3**_**SO**_**3**_ with respect to the phenyl-analogue **[3]CF**_**3**_**SO**_**3**_ but also unveiled the higher activity of **[2]CF**_**3**_**SO**_**3**_ than the reference drug cisplatin, within a tridimensional environment.

**Figure 8:**
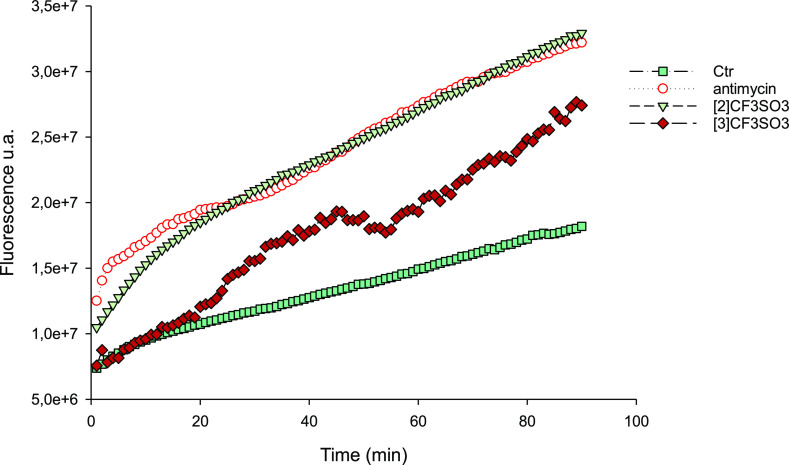
ROS production in U1285 cells. Cells were preincubated in PBS/10 mM glucose medium for 20 min at 37 °C in the presence of 10 μM CM–H_2_DCFDA and then treated with the tested compounds.

Afterward, we evaluated the ability of **[2]CF**_**3**_**SO**_**3**_ and **[3]CF**_**3**_**SO**_**3**_ to increase the total basal production of reactive oxygen species (ROS) in U1285 cancer cells. As clearly shown in Figure 8, **[2]CF**_**3**_**SO**_**3**_ triggered a substantial time-dependent rise in cellular basal hydrogen peroxide production, similar to the effect induced by antimycin, a well-known inhibitor of mitochondrial Complex III in the respiratory chain. Complex **[3]CF**_**3**_**SO**_**3**_ was also effective in enhancing cellular basal ROS production, although to a significantly lower extent.

Overall, previous findings on related diiron complexes as well as the data collected in this study suggest that the triiron complex exerts its antitumor effect by hampering cellular redox homeostasis, possibly disrupting mitochondria, and this activity is enhanced by the presence of the ferrocenyl group.^23−27^

## 3 Concluding Remarks

The ferrocene skeleton possesses distinct properties that have inspired its conjugation with other metal structures, pointing to various applications. Here, we have described the use of CNFc as a ligand for the Fe_2_Cp_2_(CO)_3_ scaffold and its subsequent methylation to generate the first ferrocenyl-decorated aminocarbyne, **[2]**^+^. Notably, the ferrocenyl substituent provides a stabilizing effect to the aminocarbyne system and the entire cationic complex, which is indefinitely air stable and exhibits a significant robustness in aqueous solutions under pseudophysiological conditions. Spectroscopic data and DFT outcomes highlight that the *N*-ferrocenyl aminocarbyne ligand behaves as a moderate π-acceptor strictly similar to its *N*-phenyl counterpart. In comparison to related diiron complexes, the triiron complex **[2]**^+^ exhibits a distinct electrochemical behavior, consisting in reduction and oxidation events centered at the [Fe^I^Fe^I^] core and the Fe^II^ of the ferrocenyl, respectively. Cellular experiments conducted on both the *N*-ferrocenyl, **[2]**^+^, and *N*-phenyl, **[3]**^+^, aminocarbyne complexes suggest that the unique combination of electrochemical properties in **[2]**^+^ is key to its enhanced ability to trigger the production of ROS within cancer cells. It is remarkable that, while **[2]**^+^ displays an averagely moderate 2D antiproliferative activity against cancer cell lines, its performance in a more reliable 3D model exceeds that of the clinical drug cisplatin. The conjugation of an easily available diiron framework with the ferrocenyl moiety represents a promising strategy with potential implications in drug development.^27^ Future studies will focus on the modification of **[2]**^+^, exploiting the arsenal of reactions documented for analogous diiron aminocarbyne complexes,^19^ and aiming to produce novel organometallic structures with, hopefully, optimized characteristics and pharmacological profiles.

## 4 Experimental Section

### 4.1 General Experimental Details

Reactants and solvents were purchased from Alfa Aesar, Merck, Strem, or TCI Chemicals and were of the highest purity available. POCl_3_ and Et_3_N (over 4 Å MS) were stored under N_2_ atmosphere as received. Syntheses were conducted under N_2_ atmosphere using standard Schlenk techniques, and products were conserved under N_2_ once isolated. Complexes **[4,5]CF**_**3**_**SO**_**3**_ were prepared according to the literature.^24^ Dichloromethane, tetrahydrofuran, and diethyl ether were dried with the solvent purification system mBraun MB SPS5, while acetonitrile was distilled from CaH_2_ and diisopropylamine from BaO. Chromatography separations were carried out on columns of silica gel (70–230 mesh), neutral alumina, or deactivated alumina (Merck, 4% w/w water) using solvents from the bottle under N_2_ flux. IR spectra of solutions were recorded using a CaF_2_ liquid transmission cell (1500–2300 cm^–1^) on a PerkinElmer Spectrum 100 FT-IR spectrometer. IR bands attributed to terminal and bridging CO/CNR ligands are indicated as *t*-CO/*t*-CN and μ-CO/μ-CN, respectively. UV–vis spectra were recorded on an Ultraspec 2100 Pro spectrophotometer using PMMA cuvettes (1 cm path length). IR and UV–vis spectra were processed with Spectragryph.^68^ NMR spectra were recorded on a Bruker Avance II DRX400 instrument equipped with a BBFO broadband probe. Chemical shifts were referenced to the residual solvent peaks^69^ or external standard (CFCl_3_ for ^19^F NMR). NMR spectra were assigned with the assistance of ^1^H NOESY, ^1^H–^1^H COSY, and ^1^H–^13^C (*gs*-HSQC and *gs*-HMBC) correlation experiments.^70^ NMR signals due to secondary isomeric forms (where it has been possible to detect them) were italicized. Elemental analyses were performed on a Vario MICRO cube instrument (Elementar).

### 4.2 Optimized Synthesis and Characterization of CNFc and Phenyl Isocyanide

#### 4.2.1 Isocyanoferrocene, CNFc (Scheme 3)

This synthesis was performed with slight modifications to previously reported procedures.^37,71,72^ Aminoferrocene was prepared using the procedure described in the Supporting Information (Scheme S1). In a Schlenk tube at 0 °C, phenyl formate (0.76 mL, 7.03 mmol) was added to aminoferrocene (707 mg, 3.51 mmol), and this mixture was stirred for 4 h. Complete consumption of aminoferrocene was checked by TLC (eluent: Et_2_O). All volatiles were removed under vacuum at 40 °C. The resulting brown oil was dissolved into a hexane/Et_2_O mixture, and this solution was passed through a SiO_2_ column. Elution with hexane was allowed to remove impurities, and then a bright-orange solution was collected using Et_2_O as eluent. Drying under vacuum afforded an orange solid corresponding to ferrocenyl formamide (FcNHCHO). The solid was dissolved in CH_2_Cl_2_ (18 mL), then POCl_3_ (0.33 mL, 3.51 mmol) and anhydrous diisopropylamine (2.48 mL, 17.6 mmol) were added. The resulting solution was stirred at 0 °C, and a color change from orange to brown was noticed along 2 h. After stirring for 6 h, the reaction mixture was quenched with 40 mL of 10% aqueous K_2_CO_3_. The organic layer was separated and washed twice with 30 mL 10% aqueous K_2_CO_3_ in air. Volatiles were removed under vacuum. The residue was dissolved in Et_2_O/hexane (1:1 v/v), and this solution was passed through a SiO_2_ column. Removal of volatiles under vacuum gave the title product a crystalline orange solid. Yield 685 mg, 92%. Anal. Calcd for C_11_H_9_FeN: C, 62.60; H, 4.30; N, 6.64. Found: C, 62.38; H, 4.28; N, 6.84. IR (CH_2_Cl_2_): ν̃/cm^–1^ = 2149w-sh, 2127s (C≡N). ^1^H NMR (CDCl_3_): δ/ppm = 4.55 (t, ^3^*J*_HH_ = 2.0 Hz, 2H, C_5_H_4_); 4.30 (s, 5H, Cp); 4.12 (t, ^3^*J*_HH_ = 2.0 Hz, 2H, C_5_H_4_).

**Scheme 3:**
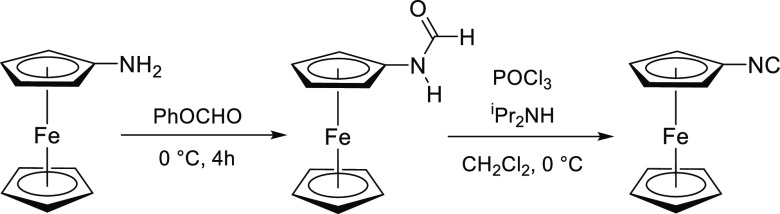
Two-step Synthesis of CNFc

#### 4.2.2 Phenyl Isocyanide, CNPh (Scheme 4)

##### 4.2.2.1 Hoffman Method^73−75^

A solution of [Bu_4_N]Br (50 mg, 0.15 mmol), aniline (490 mg, 5.3 mmol), and CHCl_3_ (0.5 mL, 6 mmol) in CH_2_Cl_2_ (5 mL) was added under vigorous stirring to a 100 mL tube containing 33% wt aqueous NaOH (2.5 g/5 mL). The biphasic mixture was stirred at ambient temperature overnight. Conversion was checked by IR, then the mixture was diluted with CH_2_Cl_2_, water, and transferred into a separatory funnel. The aqueous phase was discarded; the organic phase was extracted with saturated NaHCO_3_ (×2) and water. Volatiles were removed under vacuum without external heating (*caution! PhNC is toxic, this operation must be performed in a well-ventilated fume hood*), affording an orange-brown oil (yield: 434 mg) that was immediately allowed to react with [Fe_2_Cp_2_(CO)_4_]. Phenyl isocyanide was highly sensitive to these reaction conditions, resulting in a nonreproducible yield and purity. Silica and/or alumina chromatography resulted in its complete degradation.

**Scheme 4:**
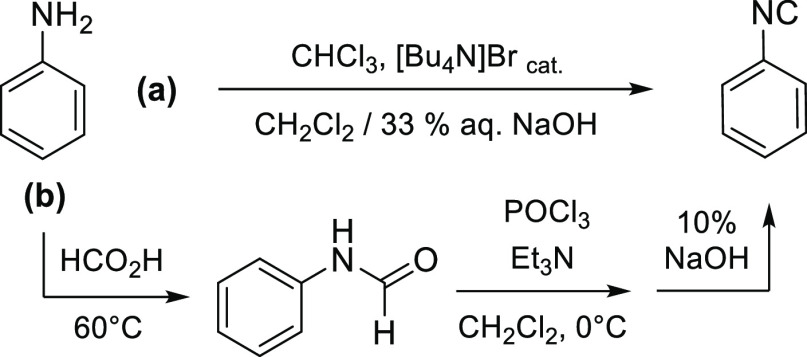
Preparation of Phenyl Isocyanide from Aniline according to the Hoffman (**a**) or Ugi (**b**) Methodologies

##### 4.2.2.2 Ugi Method

Phenyl formamide (250 mg, 2.06 mmol), prepared according to an optimized literature method^76^ (see Supporting Information), was dissolved in CH_2_Cl_2_ (6 mL) and treated with Et_3_N (0.60 mL, 4.3 mmol). The pale-yellow solution was cooled to 0 °C, and then POCl_3_ (0.20 mL, 2.1 mmol) was added dropwise. The mixture was stirred at 0 °C for 1 h, then allowed to heat to ambient temperature and treated with 10% NaOH (10 mL). The mixture was stirred for 10 min, then diluted with H_2_O and moved into a separatory funnel. The aqueous phase was extracted with CH_2_Cl_2_ (4× 10 mL). The combined organic fractions were taken to dryness under vacuum without external heating (*caution! PhNC is toxic, this operation must be performed in a well-ventilated fume hood*), affording a pale red oil that was immediately allowed to react with [Fe_2_Cp_2_(CO)_4_]. IR (CH_2_Cl_2_): υ̃/cm^–1^ = 2130s (CN), 1648m-sh, 1626m, 1589m. IR (MeCN): υ̃/cm^–1^ = 2130s (CN), 1649m-sh, 1628m, 1589m. It is essential to perform the reaction below room temperature and to quench the final mixture with NaOH; workups in less basic conditions (e.g., saturated NaHCO_3_, 10% Na_2_CO_3_)^77−79^ resulted in extensive decomposition of the isocyanide.

### 4.3 Synthesis and Characterization of Diiron Complexes

#### 4.3.1 [Fe_2_Cp_2_(CO)_2_(μ-CO){μ-CN(Me)(Fc)}]CF_3_SO_3_, **[2]CF**_**3**_**SO**_**3**_ (Figure 9)

A solution of [Fe_2_Cp_2_(CO)_4_] (430 mg, 1.21 mmol) in acetonitrile (15 mL) was treated with 1 equiv of freshly synthesized CNFc. The resulting dark-brown solution was stirred at room temperature. After 2 h, a brown precipitate was formed, stirring was prolonged for further 20 h, then volatiles were removed under reduced pressure. The resulting dark-brown solid contains [Fe_2_Cp_2_(CO)_3_(CNFc)], **1**, and a minor amount of unreacted [Fe_2_Cp_2_(CO)_4_]. IR (CH_2_Cl_2_): ν̃/cm^–1^ = 2100w (t-CN), 1988s (t-CO), 1951s (t-CO), 1788m (μ-CO), 1751 (μ-CO), 1691s (μ-CN). IR (CH_3_CN): ν̃/cm^–1^ = 2098w (t-CN), 1984s (t-CO), 1946s (t-CO), 1790m (μ-CO), 1753 (μ-CO), 1693s (μ-CN).

**Figure 9:**
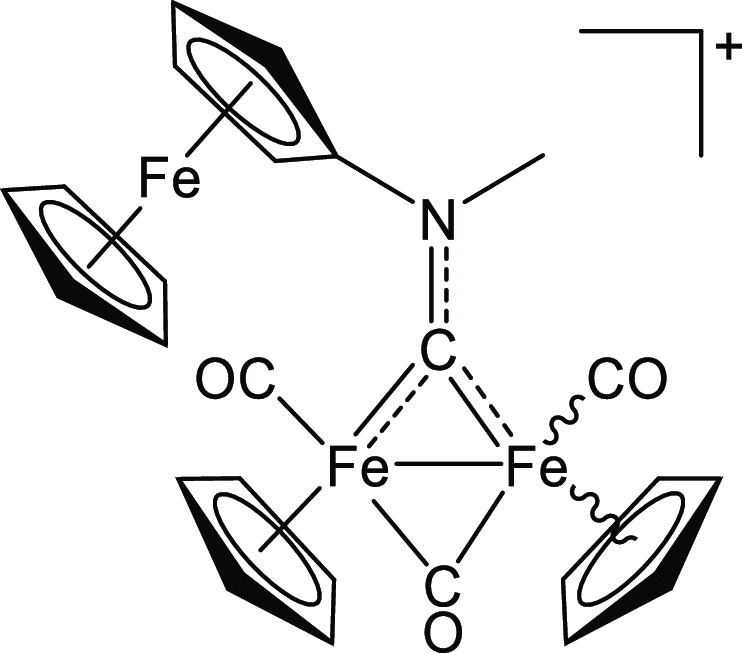
Structure of **[2]**^+^.

The residue was dissolved in CH_2_Cl_2_ (15 mL), and methyl triflate (0.13 mL, 1.2 mmol) was added dropwise to the stirred solution. The mixture was stirred at room temperature for 4 h, then it was directly charged on top of a deactivated alumina column (length 6 cm and diameter 3.5 cm). Impurities were removed by using CH_2_Cl_2_ and CH_2_Cl_2_/THF (1:1 v/v) as eluents. A bright-red band corresponding to **[2]CF**_**3**_**SO**_**3**_ was collected by using neat CH_3_CN as eluent. According to ^1^H NMR analyses, the eluted mixture was progressively enriched with the cis isomer and the final portion of the band contained pure cis-**[2]CF**_**3**_**SO**_**3**_. Solvent removal under reduced pressure allowed to obtain a red solid, which was washed with Et_2_O and dried under vacuum. Yield 744 mg, 88%. Soluble in DMSO, EtOH, CH_2_Cl_2_, acetone, insoluble in Et_2_O. X-ray quality crystals of **[2]CF**_**3**_**SO**_**3**_ were obtained CH_2_Cl_2_/pentane at −20 °C. Anal. Calcd for C_26_H_22_F_3_Fe_3_NO_6_S: C, 44.54; H, 3.16; N, 2.00; S, 4.57. Found: C, 44.38; H, 3.21; N, 1.96; S, 4.62. IR (CH_2_Cl_2_): ν̃/cm^–1^ = 2024 (s, CO); 1992 (w-m, CO); 1837 (m, μ-CO); 1527 (w-m, μ-CN). ^1^H NMR (CDCl_3_, cis/trans ratio = 5): δ/ppm = 5.43, 4.71, 4.64, 4.49, 4.41, 4.32, 4.20 (m, 4H, C_5_H_4_); 5.36, 5.23, 4.84, 4.64 (s, 10H, Cp); 4.92, 4.89 (s, 3H, Me); 4.56, 4.43 (s, 5H, Cp^Fc^). ^13^C{^1^H} NMR (CDCl_3_): δ/ppm = 326.8 (μ-CN); 255.3 (μ-CO); 209.1, 207.9 (CO); 121.0 (d, ^1^*J*_CF_ = 321 Hz, CF_3_); 112.1 (NC^Cp^); 91.7, 90.53, 90.48 (Cp); 70.8, 70.5 (Cp^Fc^); 67.7, 67.5, 66.3, 65.6, 65.4, 65.2 (C_5_H_4_); 60.5, 59.0 (Me). ^19^F{^1^H} NMR (CDCl_3_): δ/ppm = −78.1.

### 4.3.2 [Fe_2_Cp_2_(CO)_2_(μ-CO){μ-CN(Me)(Ph)}]CF_3_SO_3_, **[3]CF**_**3**_**SO**_**3**_ (Figure 10)

In a 100 mL round-bottom flask under N_2_, freshly prepared PhNC (ca. 2 mmol) was dissolved in anhydrous MeCN (20 mL) and treated with [Fe_2_Cp_2_(CO)_4_] (730 mg, 2.06 mmol). The dark red suspension was stirred at room temperature overnight; a dark violet shade appeared in less than 1 h. Conversion was checked by IR (MeCN), and then volatiles were removed under vacuum. The dark violet residue was suspended in Et_2_O and treated with 48% aq HBF_4_ (0.3 mL, 2.3 mmol), affording a scarlet red solid. The suspension was stirred for 2 h then moved on top of an alumina column (h 5 d 3.4 cm). A dark red band containing [Fe_2_Cp_2_(CO)_4_] and other impurities was eluted using Et_2_O, then a dark violet band was eluted using a 5% Et_3_N solution in CH_2_Cl_2_ (ca. 200 mL). Volatiles were removed under vacuum, affording a dark violet residue containing [Fe_2_Cp_2_(CO)_2_(μ-CO)(μ-CNPh)]^80^ {IR (CH_2_Cl_2_): υ̃/cm^–1^ = 1991s (t-CO), 1951s (t-CO), 1781m (μ-CO), 1691s (μ-CN)} and a minor amount of [Fe_2_Cp_2_(CO)(CNPh)(μ-CO)(μ-CNPh)]^52^ {IR (CH_2_Cl_2_): υ̃/cm^–1^ = 2093s (t-CN)}. The solid (ca. 0.8 mmol) was dissolved in anhydrous CH_2_Cl_2_ (15 mL), and methyl triflate (0.10 mL, 0.91 mmol) was added dropwise under stirring. The dark violet solution rapidly changed to dark red and was stirred at room temperature for 4 h. Next, the mixture was moved on top of an alumina column (h 4.5, d 4.5 cm). Impurities were eluted with CH_2_Cl_2_, CH_2_Cl_2_/THF 1:1 v/v, and THF, then a red band was eluted with MeCN (ca. 200 mL). Volatiles were removed under vacuum. The residue was dissolved in CH_2_Cl_2_ and filtered over a Celite pad. The filtrate solution was taken to dryness under vacuum, affording a red foamy solid that was washed with hexane, dried under vacuum (40 °C), and stored under N_2_. Yield: 194 mg, ca. 41%. Soluble in CH_2_Cl_2_, acetone, EtOH, and DMSO, scarcely soluble in Et_2_O, toluene, insoluble in water. Anal. Calcd for C_22_H_18_F_3_Fe_2_NO_6_S: C, 44.55; H, 3.06; N, 2.36; S, 5.41. Found: C, 44.78; H, 3.20; N, 2.55; S, 5.24. IR (CH_2_Cl_2_): υ̃/cm^–1^ = 2025s (CO), 1993w-sh (CO), 1839m (μ-CO), 1549m, 1531w-sh (CN). ^1^H NMR (CDCl_3_): δ/ppm = 7.76 (br., 2H, Ph^ortho^); 7.62 (t, ^3^*J*_HH_ = 7.5 Hz, 2H, Ph^meta^); 7.54 (t, ^3^*J*_HH_ = 7.3 Hz, 1H, Ph^para^); 5.43 (s, 5H, Cp); 4.72 (s, 5H, Cp′); 4.55 (s, 3H, Me). ^13^C{^1^H} NMR (CDCl_3_): δ/ppm = 324.5 (μ-CN); 254.4 (μ-CO); 208.8, 207.7 (CO); 150.6 (Ph^ipso^); 130.7 (Ph^ortho^); 129.6 (Ph^meta^); 125.3 (Ph^para^); 120.9 (d, ^1^*J*_CF_ = 309 Hz, CF_3_); 90.5, 90.2 (Cp); 57.3 (Me). ^19^F{^1^H} NMR (CDCl_3_): δ/ppm = −78.0. The isolated compound contains ca. 7% mol. of [Fe_2_Cp_2_(CO)(CNPh)(μ-CO){μ-CN(Me)(Ph)}]CF_3_SO_3_. ^1^H NMR (CDCl_3_): δ/ppm = 7.48 (m), 7.10 (d) (Ph); 5.34 (s, 5H, Cp); 4.69 (s, 5H, Cp′); 4.55 (s, 3H, NMe). IR (CH_2_Cl_2_): υ̃/cm^–1^ = 2127 (t-CN), 1773 (μ-CO).

**Figure 10:**
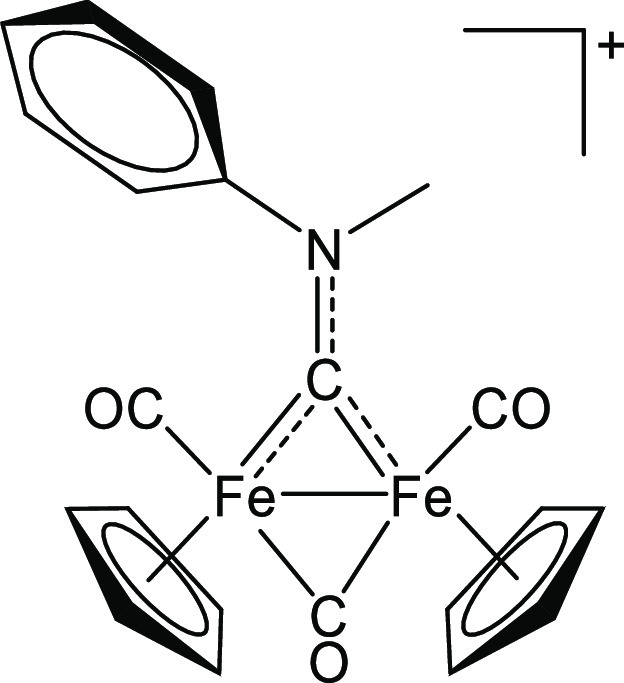
Structure of **[3]**^+^.

### 4.4 Isomerization Reactions of **[2]CF**_**3**_**SO**_**3**_

General procedure: Complex **[2]CF**_**3**_**SO**_**3**_ (ca. 15 mg) was heated at reflux under N_2_ in selected solvents for several hours, then the solvent was removed under vacuum, and the residue was analyzed by ^1^H NMR (CDCl_3_ solution). Thermal treatment in isopropanol, acetonitrile, methanol, or toluene (1–4 h) did not affect the isomeric ratio. Refluxing in DMF resulted in extensive degradation. Conditions leading to an increased isomeric ratio are reported in Table 5. Variable byproduct formation and/or degradation were observed, generally more pronounced as the isomeric ratio increased.

**Table 5:** Experimental Conditions and Resulting Isomeric Ratios (Evaluated by Subsequent ^1^H NMR Spectra) Related to **[2]CF**_**3**_**SO**_**3**_

solvent, time, temperature	cis/trans ratio
EtOH, 4 h, reflux	6
EtOH, 7 h, reflux	8
acetone, 2.5 h, reflux	18
DMF, 4 h, 110°C	9
DMF, overnight, 110°C	17

### 4.5 Electrochemistry

CV measurements were performed with a PalmSens4 instrument interfaced to a computer employing PSTrace5 electrochemical software. Anhydrous CH_2_Cl_2_ (Merck) and THF (Merck) were stored under argon over 3 Å molecular sieves. [N^*n*^Bu_4_]PF_6_ (Fluka, electrochemical grade) and FeCp_2_ (Fluka) were used without further purification. CV measurements were carried out under argon using 0.2 M [N^*n*^Bu_4_]PF_6_ in CH_2_Cl_2_ or THF as the supporting electrolyte. The working and the counter electrodes consisted of a Pt disk and a Pt gauze, respectively. A leakless miniature Ag/AgCl/KCl (3.4 mol/L) electrode (eDAQ) was employed as a reference. The three-electrode home-built cell was predried by heating under vacuum and filled with argon. The Schlenk-type construction of the cell maintained anhydrous and anaerobic conditions. The solution of the supporting electrolyte, prepared under argon, was introduced into the cell, and the CV of the solvent was recorded. The analyte was then introduced, and voltammograms were recorded; lastly, a small amount of ferrocene was added, and the CV was repeated. Under the present experimental conditions, the one-electron oxidation of ferrocene occurred at *E*° = +0.45 V vs Ag/AgCl in CH_2_Cl_2_, and at *E*° = +0.57 V vs Ag/AgCl in THF.

PB solution (Na_2_HPO_4_/KH_2_PO_4_, Σ*c*_(PO_4_)_ = 50 mM, pH = 7.3) was prepared in ultrapure H_2_O and used as an aqueous supporting electrolyte. The three-electrode home-built cell was equipped with a Pt sheet counter electrode, a Teflon-encapsulated glassy-carbon (GC) working electrode (BASi, ø 3 mm), and a leak-free Ag/AgCl/KCl (3.4 mol/L) reference electrode (eDAQ). Prior to measurements, the working GC electrode was polished by the following procedure:^81^ manual rubbing with 0.3 μM Al_2_O_3_ slurry in water (eDAQ) for 2 min, then sonication in ultrapure water for 10 min, manual rubbing with 0.05 μM Al_2_O_3_ slurry in water (eDAQ) for 2 min, then sonication in ultrapure water for 10 min. The PB (5.0 mL) was introduced into the cell, deaerated by argon bubbling for some minutes, and the CV of the solvent recorded. The analyte was then introduced, and voltammograms were recorded (scan rate: 0.1 V/s). Under the present experimental conditions, the one-electron reduction of ferricinium in the PB occurred at *E*° = +0.20 V vs Ag/AgCl.

Controlled potential coulometry was performed in an H-shaped cell with anodic and cathodic compartments separated by a sintered-glass disk. The working macroelectrode and counter-electrode were platinum gauze.

IR SEC measurements were carried out using an OTTLE cell equipped with CaF_2_ windows, platinum mini-grid working and auxiliary electrodes, and a silver wire pseudoreference electrode.^57^ During the microelectrolysis procedures, the electrode potential was controlled by a PalmSens4 instrument interfaced to a computer employing PSTrace5 electrochemical software. Argon-saturated CH_2_Cl_2_ or THF/[N^*n*^Bu_4_]PF_6_ 0.2 M solutions of the analyzed compound were used. The in situ SEC experiments were performed by collecting IR spectra at fixed time intervals during oxidation or reduction, obtained by continuously increasing or lowering the initial working potential at a scan rate of 1.0 or 2.0 mV s^–1^, or by electrolysis at constant potential. In this second procedure, during the electrolysis, the IR spectra were collected each 30 s.

### 4.6 DFT Calculations

Geometry optimizations were performed using the PBEh-3c method, which is a reparametrized version of PBE0^82^ (with 42% HF exchange) that uses a split-valence double-ζ basis set (def2-mSVP)^83,84^ and adds three corrections considering dispersion, basis set superposition, and other basis set incompleteness effects.^85−87^ The C-PCM implicit solvation model was added to PBEh-3c calculations.^88,89^ IR simulations were carried out using the harmonic approximation, from which zero-point vibrational energies and thermal corrections (*T* = 298.15 K) were obtained.^90^ The software used was ORCA version 5.0.3.^91^ The output was elaborated using MultiWFN, version 3.8.^92^ The cartesian coordinates of the DFT-optimized structures were collected in a separated.xyz file.

### 4.8 Behavior in Aqueous Solutions

#### 4.8.1 Solubility in D_2_O

The selected diiron complex was suspended in a D_2_O solution (0.7 mL) containing dimethyl sulfone (Me_2_SO_2_; 4.5 × 10^–3^ M), and this suspension was stirred at room temperature (21 ± 1 °C) for 3 h. The saturated solution was filtered over Celite and analyzed by ^1^H NMR (delay time = 3 s; number of scans = 20). The concentration (=solubility) was calculated as the relative integral with respect to Me_2_SO_2_ as an internal standard [δ/ppm = 3.14 (s, 6H)] (Table 2). NMR data are reported in the Supporting Information.

#### 4.8.2 Octanol/Water Partition Coefficient (Log *P*_ow_)

Partition coefficients (*P*_ow_), defined as *P*_ow_ = *c*_org_/*c*_aq_, where *c*_org_ and *c*_aq_ were the molar concentrations of the selected compound in the *n*-octanol and aqueous phases, respectively, were determined by the shake-flask method and UV–vis measurements, according to a previously described procedure.^93,94^ All operations were carried out at room temperature (21 ± 1 °C). The stock solution of **[2]CF**_**3**_**SO**_**3**_ was prepared in water-saturated octanol, while the stock solution of **[2]CF**_**3**_**SO**_**3**_ in octanol-saturated water. The wavelength corresponding to a well-defined maximum of shoulder absorption of each compound (320–400 nm range) was used for UV–vis quantitation. The procedure was repeated three times on each sample (from the same stock solution); the results are given as the mean ± standard deviation (Table 2).

#### 4.8.3 Stability in CD_3_OD/D_2_O Mixture

The selected diiron complex (ca. 5 mg) was dissolved in CD_3_OD/D_2_O (5:2 v/v, ca. 0.7 mL) containing 4.03 × 10^–3^ M dimethylsulfone (Me_2_SO_2_) as an internal standard. This solution was filtered over Celite, then transferred into an NMR tube, and the ^1^H NMR spectrum was recorded. The mixture was maintained at 37 °C for 48 h. After filtration over Celite, the solution was analyzed by ^1^H NMR. The residual amount of starting material in the final solution (with respect to the initial spectrum) was calculated as the relative integral with respect to Me_2_SO_2_ as an internal standard. NMR spectra were recorded using the following settings: number of scans = 20; relaxation delay = 3 s **[2]CF**_**3**_**SO**_**3**_. ^1^H NMR (D_2_O/CD_3_OD 5:2 v/v): δ/ppm = 5.38, 5.31 (s, 5H); 4.94 (s, *), 4.80 (s, *), 4.78 (s, *); 4.60 (s), 4.57–4.54 (m), 4.51 (s) (7H); cis/trans ratio ca. 1. *Over HDO peak. **[3]CF**_**3**_**SO**_**3**_. ^1^H NMR (D_2_O): δ/ppm = 7.74–7.62 (m, 5H), 5.44 (s, 5H), 4.55 (s, 3H). Cp′ is hidden by the HDO peak. ^1^H NMR (D_2_O/CD_3_OD 5:2 v/v): δ/ppm = 7.85–7.59 (m, 5H), 5.46 (s, 5H), 4.78 (s), 4.56 (s, 3H).

#### 4.8.4 Stability in CD_3_OD/DMEM Mixture

Deuterated cell culture medium (DMEM-d) was prepared by dissolving powdered DMEM cell culture medium (1000 mg/L glucose and l-glutamine, without sodium bicarbonate and phenol red; D2902-Sigma-Aldrich) in D_2_O (10 mg/mL, according to the manufacturer’s instructions). The solution was treated with Me_2_SO_2_ (ca. 6 × 10^–3^ M), NaH_2_PO_4_, and Na_2_HPO_4_ (25 mM total phosphate, pD = 7.4),^95^ then stored under N_2_. Solutions of diiron complexes in a DMEM-*d*/CD_3_OD 5:2 v/v mixture were prepared and treated as described above. The residual amount of starting material in solution after 24 h at 37 °C was calculated with respect to Me_2_SO_2_ as an internal standard (Table 2).

### 4.9 Biological Studies

#### 4.9.1 Cytotoxicity

The tested complexes were dissolved in the minimum DMSO amount prior to cell culture testing. A calculated amount of the stock drug DMSO solution was added to the cell culture media to reach a final maximum DMSO concentration of 0.5%, which had no effects on cell viability. Cisplatin was dissolved in a 0.9% sodium chloride solution. MTT [3-(4,5-dimethylthiazol-2-yl)-2,5-diphenyltetrazolium bromide], cisplatin, and ImmunoPure *p*-nitrophenyl phosphate (APH) were obtained from Sigma Chemical Co., St. Louis, USA.

#### 4.9.2 Cell Cultures

Human colon (HCT-15) and pancreatic (PSN-1) carcinoma cell lines, along with human SCLC (U1285), were obtained from the American Type Culture Collection (ATCC, Rockville, MD). Human ovarian 2008 cancer cells were kindly provided by Prof. G. Marverti (Dept. of Biomedical Science of Modena University, Italy). Human cervical A431 cancer cells were kindly provided by Prof. P. Perego (Fondazione IRCCS Istituto Nazionale Tumori, Milan, Italy).

Cell lines were maintained in the logarithmic phase at 37 °C in a 5% carbon dioxide atmosphere using RPMI-1640 culture medium containing 10% fetal calf serum (Euroclone, Milan, Italy), antibiotics (50 units/mL penicillin and 50 μg/mL streptomycin), and 2 mM l-glutamine.

#### 4.9.3 Spheroid Cultures

Spheroid cultures were obtained by seeding 2.5 × 10^3^ cells/well in a round-bottom nontissue culture-treated 96 well-plate (Greiner Bio-one, Kremsmünster, Austria) in phenol red free RPMI-1640 medium (Sigma Chemical Co.), containing 10% FCS and supplemented with 20% methyl cellulose stock solution.

#### 4.9.4 MTT Assay

The growth inhibitory effect toward tumor cells was evaluated by means of an MTT assay, as previously described.^96^ IC_50_ values were calculated with a four-parameter logistic (4-PL) model. All the values were the means ± SD of not less than four independent experiments.

#### 4.9.5 Acid Phosphatase Assay

An APH-modified assay was used for determining cell viability in 3D spheroids, as previously described.^97^ IC_50_ values were calculated with a four-parameter logistic (4-PL) model. All the values are the means ± SD of not less than four independent experiments.

#### 4.9.6 ROS Production

The production of ROS was measured in U1285 cells (10^4^ per well) grown for 24 h in a 96-well plate in RPMI medium without phenol red (Sigma Chemical Co.). Cells were then washed with PBS and loaded with 10 μM 5-(and-6)-chloromethyl-2′,7′-dichlorodihydrofluorescein diacetate acetyl ester (CM–H_2_DCFDA) (Molecular Probes-Invitrogen, Eugene, OR) for 25 min, in the dark. Afterward, cells were washed with PBS and incubated with increasing concentrations of the tested compounds. Fluorescence increase was estimated utilizing the wavelengths of 485 nm (excitation) and 527 nm (emission) in a VICTOR X3 (PerkinElmer, USA) plate reader. Antimycin (3 μM, Sigma Chemical Co.), a potent inhibitor of Complex III in the electron transport chain, was used as a positive control.

## Supporting Information Available

Synthesis/characterization of CNFc; IR and NMR spectra; computational results; spectroelectrochemical studies (PDF)
Cartesian coordinates of the DFT-optimized structures (XYZ)

## Supplementary Material

ic3c03408_si_001.pdf

ic3c03408_si_002.xyz
