# Supplementary material for: Triiron Complex with N-Ferrocenyl Aminocarbyne Ligand Bridging a Diiron Core: DFT, Electrochemical, and Biological Insights
Source: Inorg Chem. 2024 Jan 3;63(2):1054–67. doi: 10.1021/acs.inorgchem.3c03408 (PMC10792607; doi:10.1021/acs.inorgchem.3c03408)
Supplement: Supplementary file 1 — ic3c03408_si_001.pdf [file ic3c03408_si_001.pdf]

# Supporting Information

## A Triiron Complex with *N*-Ferrocenyl Aminocarbyne Ligand Bridging a Diiron Core: DFT, Electrochemical and Biological Insights

Chiara Saviozzi,[a] Lorenzo Biancalana,[a] Tiziana Funaioli,[a] Marco Bortoluzzi,[b] Michele De Franco,[c] Massimo Guelfi,[a] Valentina Gandin,[c] Fabio Marchetti [a,*]

[a] University of Pisa, Department of Chemistry and Industrial Chemistry, Via G. Moruzzi 13, I-56124 Pisa, Italy.

[b] University of Venezia "Ca' Foscari", Department of Molecular Science and Nanosystems, Via Torino 155, I-30170 Mestre (VE), Italy.

[c] University of Padova, Department of Pharmaceutical and Pharmacological Sciences, Via F. Marzolo 5, I-35131 Padova, Italy.

**Corresponding Author**

*E-mail address: fabio.marchetti@unipi.it; Webpage: https://people.unipi.it/fabio_marchetti/

<u>**Table of contents**</u> *Pages*

Synthesis of aminoferrocene and phenyl formamide S2

**Figures S1-S2.** Characterization of ferrocenyl isocyanide S5

**Figure S3**. Possible isomeric forms of **1** S6

**Figures S4-S14**. IR and NMR spectra of diiron / triiron compounds S7-S12

**Figure S15**. DFT optimized geometries of $[Fe_2Cp_2(CO)_2(\mu\text{-CO})(\mu\text{-CNPh})]$ S13

**Figure S16**. Simulated IR spectra S14

**Figure S17-S21**. Spectro-electrochemical studies and CV S15-S17

**Figure S22**. Simulated IR spectra S19

**References** S20

## Synthesis of aminoferrocene, $NH_2Fc$ (Scheme S1)

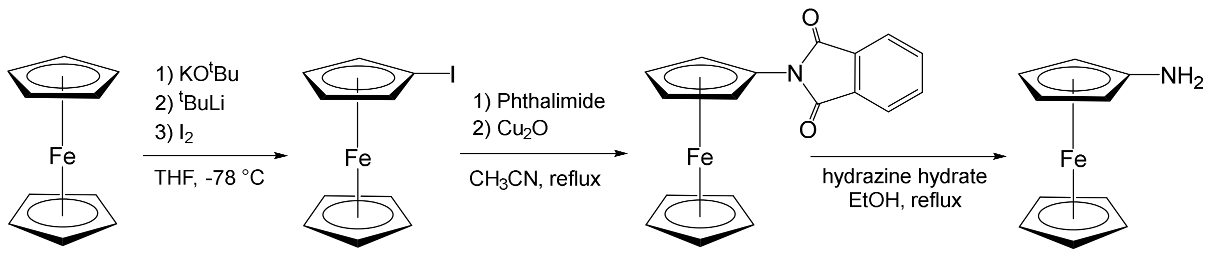


**Scheme S1**. Three-step synthesis of aminoferrocene

Aminoferrocene was synthesized by combining previously reported procedures,[1,2,3] with a focus on optimizing yields, reaction times, and the use of safer reagents for a gram scale preparation. In a three-neck flask, under $N_2$ atmosphere, $^t$BuLi (12.5 mL of 1.7 M pentane solution, 21.5 mmol) was added to a cooled (-78 °C) solution of ferrocene (4.00 g, 21.5 mmol) and KO$^t$Bu (0.29 g, 2.6 mmol) in THF (50 mL), over 20 min under vigorous stirring via isobaric dropping funnel. The mixture was stirred for a further 1 h at -78 °C. Hence, $I_2$ (6.85 g, 27.0 mmol) was added in one portion, and the resulting mixture was allowed to warm to room temperature and subsequently stirred overnight. The obtained mixture was diluted with $Et_2O$ (100 mL) and then washed with a saturated solution of $Na_2S_2O_3$ (4 x 50 mL) and then with $H_2O$ (2 x 50 mL). The collected organic phase was dried over $Na_2SO_4$ and isolated. The volatile components were evaporated under vacuum, affording a brown sticky solid (iodoferrocene), whose identity was checked by $^1$H NMR.[2,4] Yield 4.24 g, 62%. The residual presence of unreacted ferrocene did not affect the subsequent steps, thus the crude product was used without further purification.

Next, a solution of iodoferrocene (3.0 g, 9.6 mmol), phthalimide (1.76 g, 12.0 mmol) and $Cu_2O$ (1.72 g, 12.0 mmol) in $CH_3CN$ (50 mL) was refluxed for 24 h in a Schlenk flask. Afterwards, volatiles were evaporated under reduced pressure, and the resulting residue was dissolved in ethyl acetate (100 mL). This solution was filtered through a celite pad. The filtrate was washed with NaOH 2M (2 x 100 mL) and brine (2 x 100mL), and subsequently dried over $Na_2SO_4$. The resultant solution was concentrated under reduced pressure. Purification was performed by flash chromatography on silica gel. A red fraction corresponding to *N*-ferrocenyl phthalimide was

collected using an ethyl acetate/heptane mixture (1:9 v/v). Yield 2.48 g, 78 %. $^1$H NMR spectrum was consistent with the literature.[1]

Next, deaerated hydrazine monohydrate (15 mL) was added to a suspension of *N*-ferrocenyl phthalimide (2.48 g, 7.47 mmol) in deaerated ethanol (50 mL) in a Schlenk flask, and the resulting mixture was refluxed for 4 h. The reaction mixture was subsequently cooled to 0 °C, and deaerated water (100 mL) was added. The resulting mixture was extracted with $Et_2O$ (4 x 50 mL), and the organic phase was dried over $Na_2SO_4$. The solvent was evaporated under vacuum, thus aminoferrocene was obtained as yellow solid. Yield 1.20 g, 80 %. $^1$H NMR spectrum was consistent with the literature.[2]

## Synthesis of phenyl formamide (Scheme S2)

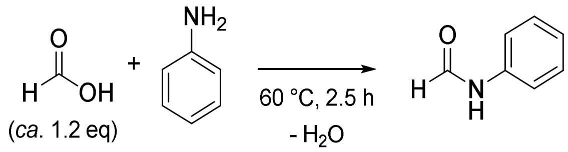


**Scheme S2**. Synthesis of phenyl formamide

Herein we report an optimized, 15 g-scale synthesis of phenyl formamide based on the procedure of Bhanange and co-workers.[5] In a 50 mL round bottom flask, 98 % formic acid (6 mL, *ca*. 156 mmol) was introduced and heated at 60 °C. Aniline (12 mL, 132 mmol) was rapidly added to the hot solution under vigorous stirring. The mixture was kept at 60 °C for 2.5 h then diluted with $Et_2O$ (20 mL) and moved into a separatory funnel. The organic solution was extracted with a saturated $NaHCO_3$ solution (30 mL; *caution! considerable $CO_2$ evolution inside the funnel*) then water (3 x 30 mL). Volatiles were removed under vacuum from the $Et_2O$ solution, affording a pale red oil. The residue was dissolved in $CH_2Cl_2$ and moved on top of a silica column (h 7 cm, d 5.3 cm). Impurities were eluted with $CH_2Cl_2$, then a pale yellow band was eluted with $Et_2O$. The eluate was taken to dryness under vacuum, affording an oil that was cooled to – 20 °C for 2-3 h to allow solidification. The resulting pale yellow waxy solid was triturated, dried under vacuum and stored at 4 °C [melting point ≈ 50 °C]. Yield: 14.14 g, 88 %. $R_f$ = 0.1 ($CH_2Cl_2$), 0.4 ($CH_2Cl_2$/$Et_2O$ 9:1 *v*/*v*), 0.7 ($Et_2O$). $^1$H NMR ($CDCl_3$): δ/ppm = 8.69 (d, $^3J_{HH}$ = 11.4 Hz), 8.39 (d, $^3J_{HH}$ = 1.4 Hz) (1H, HCO); 7.76 (s-br, NH); 7.57–7.51 (m, 1H), 7.40–7.32 (m, 2H), 7.24–7.05 (m, 2H) (Ph). $^{13}$C{$^1$H} NMR ($CDCl_3$): δ/ppm = 163.2, 159.4 (CO); 137.0, 136.8 ($Ph_{ipso}$); 129.9, 129.2 ($Ph_{meta}$); 125.5, 125.0 ($Ph_{para}$); 120.2, 119.0 ($Ph_{ortho}$).

## Characterization of ferrocenyl isocyanide

**Figure S1.** IR spectrum (1500-2200 cm$^{-1}$, $CH_2Cl_2$) of CNFc.

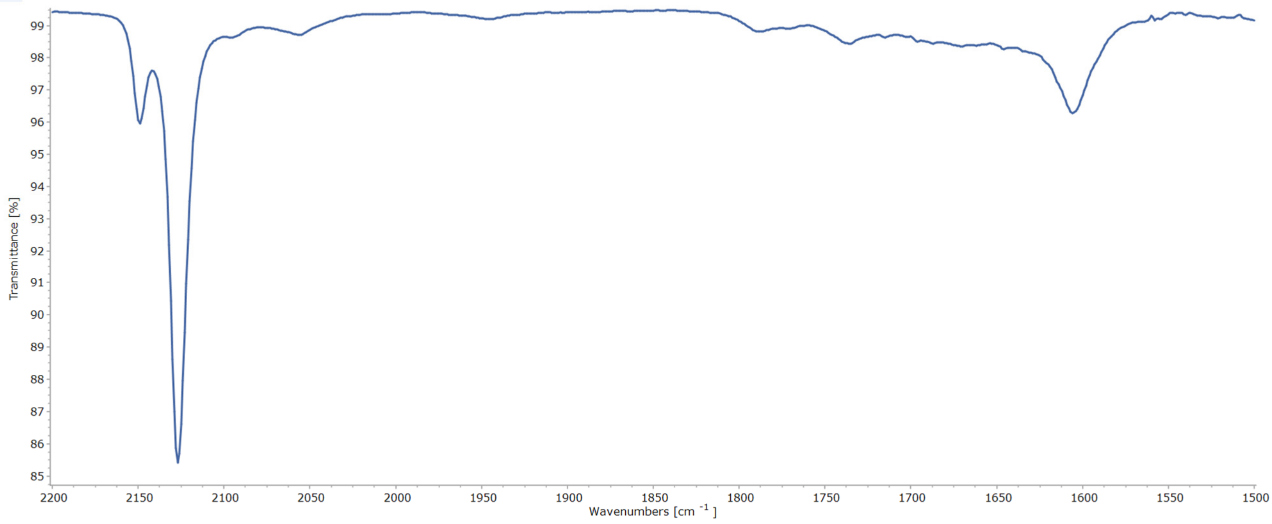


**Figure S2.** $^{1}H$ NMR spectrum (401 MHz, $CDCl_3$) of CNFc.

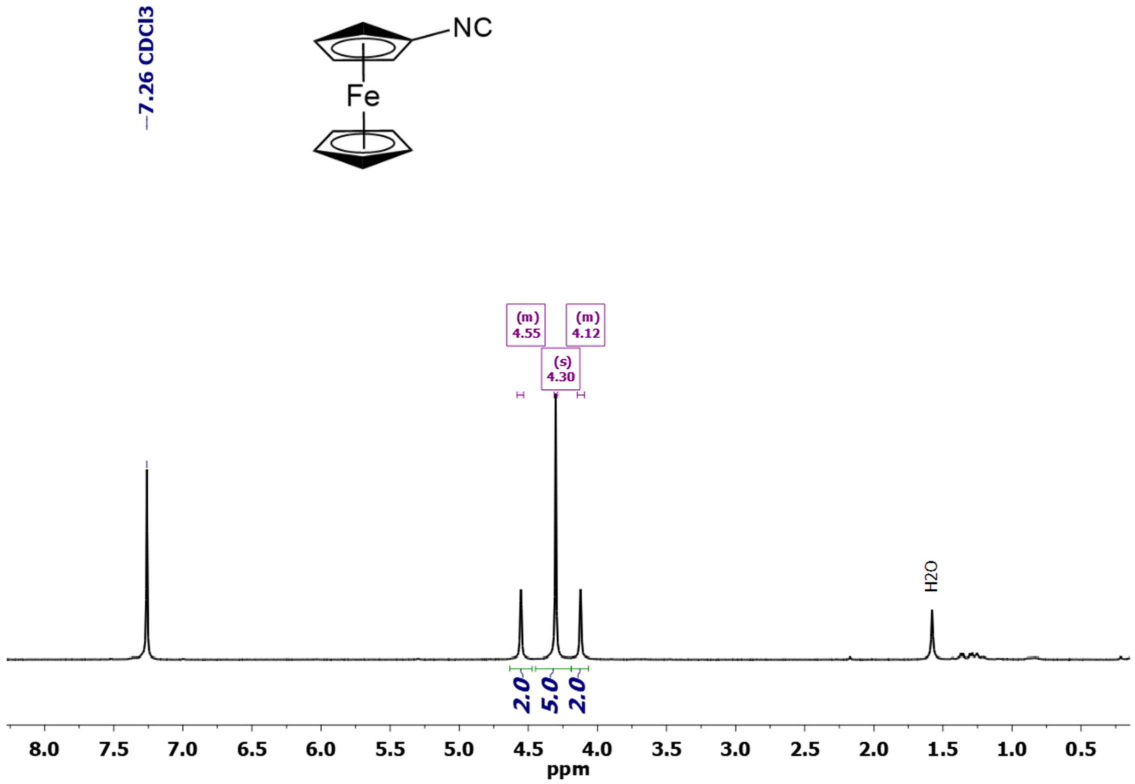

**Figure S3.** Possible isomeric forms displayed by [$Fe_2Cp_2(CO)_3(CNFc)$], **1**, according to IR spectroscopy.

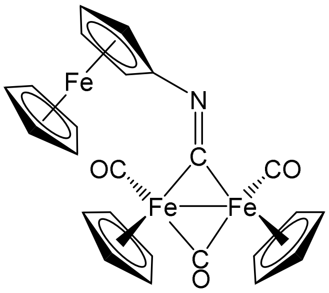

**cis-1-b**

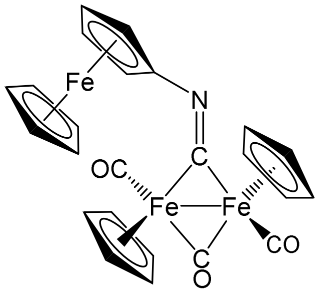

**trans-1-b**

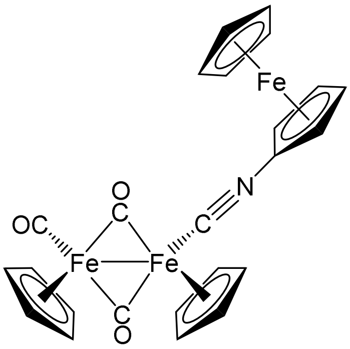

**cis-1-t**

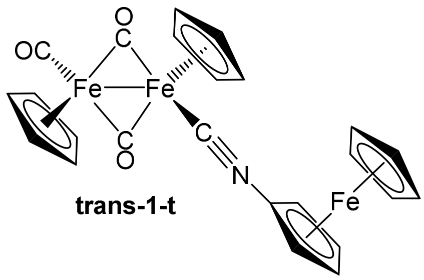

**trans-1-t**

## IR and NMR spectra of diiron / triiron compounds

**Figure S4.** Comparative view of IR spectra (1500-2200 $cm^{-1}$) of **1** in $CH_2Cl_2$ (orange line) and MeCN (cyan line) solution, in admixture with a minor amount of $[Fe_2Cp_2(CO)_4]$.

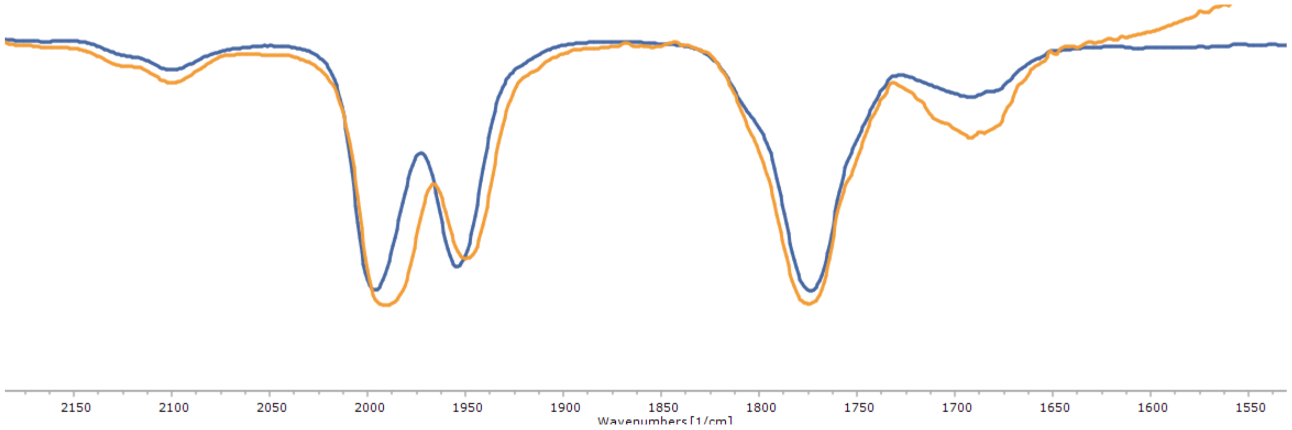


**Figure S5.** Comparative view of IR spectra (1500-2200 $cm^{-1}$, $CH_2Cl_2$) of $[Fe_2Cp_2(CO)_2(\mu\text{-}CO)\{\mu\text{-}CN(Me)(Fc)\}]CF_3SO_3$, **[2]$CF_3SO_3$**, as a 5:1 *cis*/*trans* mixture (cyan line) and as pure *cis* isomer (orange line). The pure *cis* isomer was serendipitously isolated by alumina chromatography in a non-reproducible way.

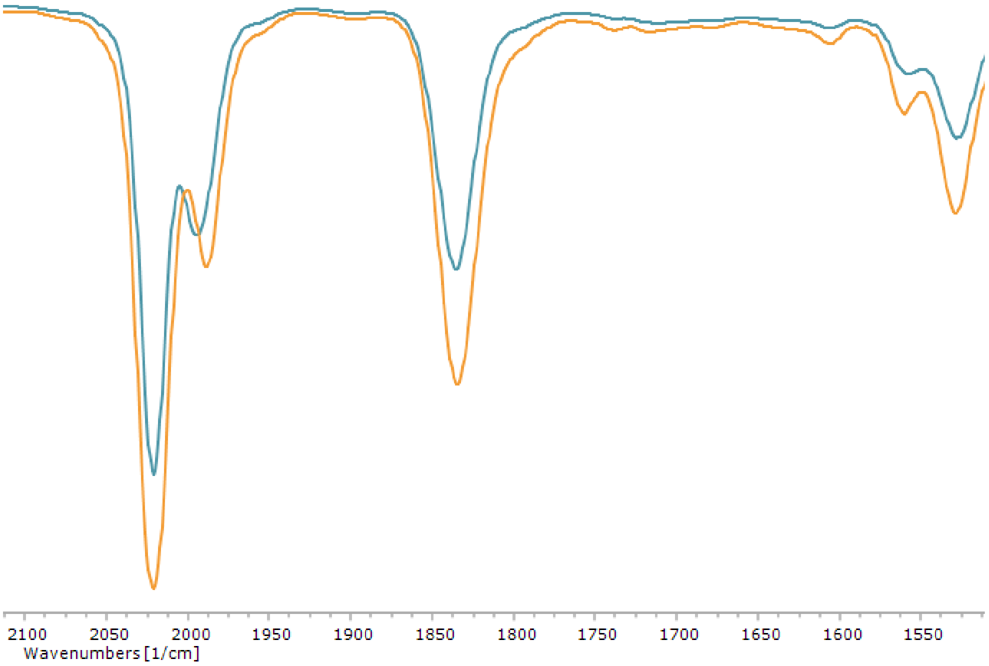

**Figure S6.** $^{1}$H NMR spectrum (401 MHz, $CDCl_3$) of **[2]CF$_3$SO$_3$** (*cis*/*trans* ratio 5:1). Resonances of the *cis* isomer are highlighted.

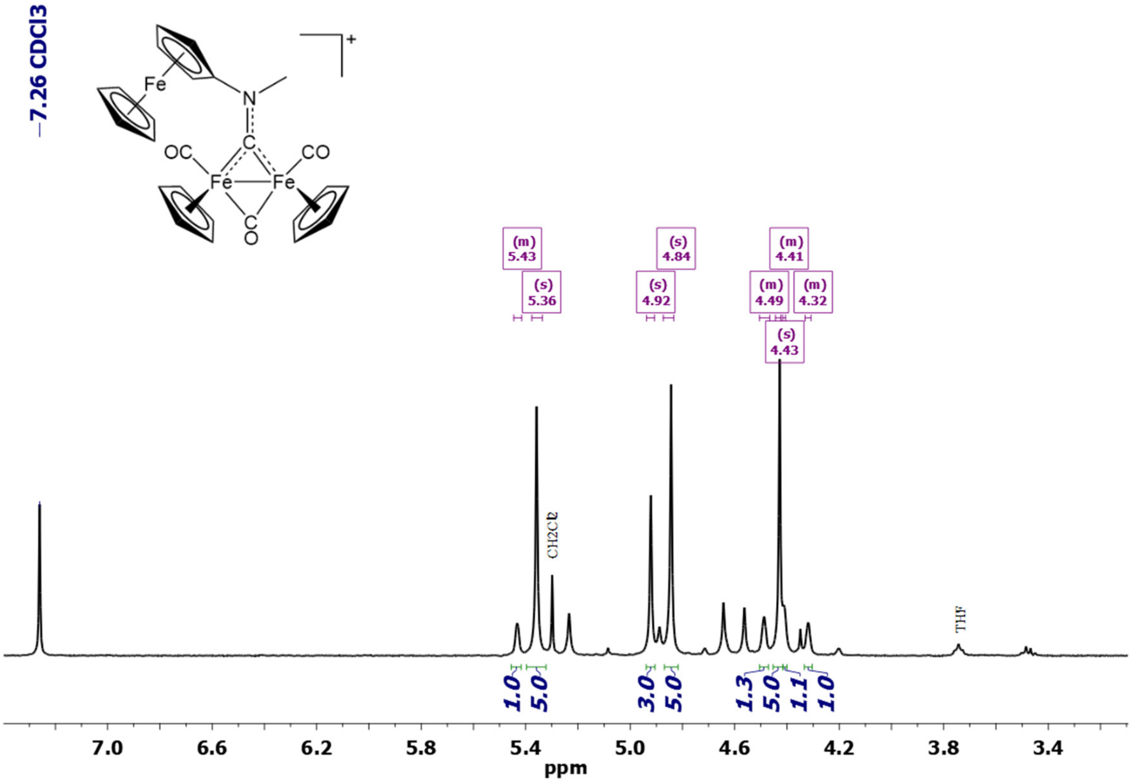


**Figure S7.** $^{1}$H NMR spectrum (401 MHz, $CDCl_3$) of **[2]CF$_3$SO$_3$** (*cis*/*trans* ratio 5:1). Resonances of the *trans* isomer are highlighted.

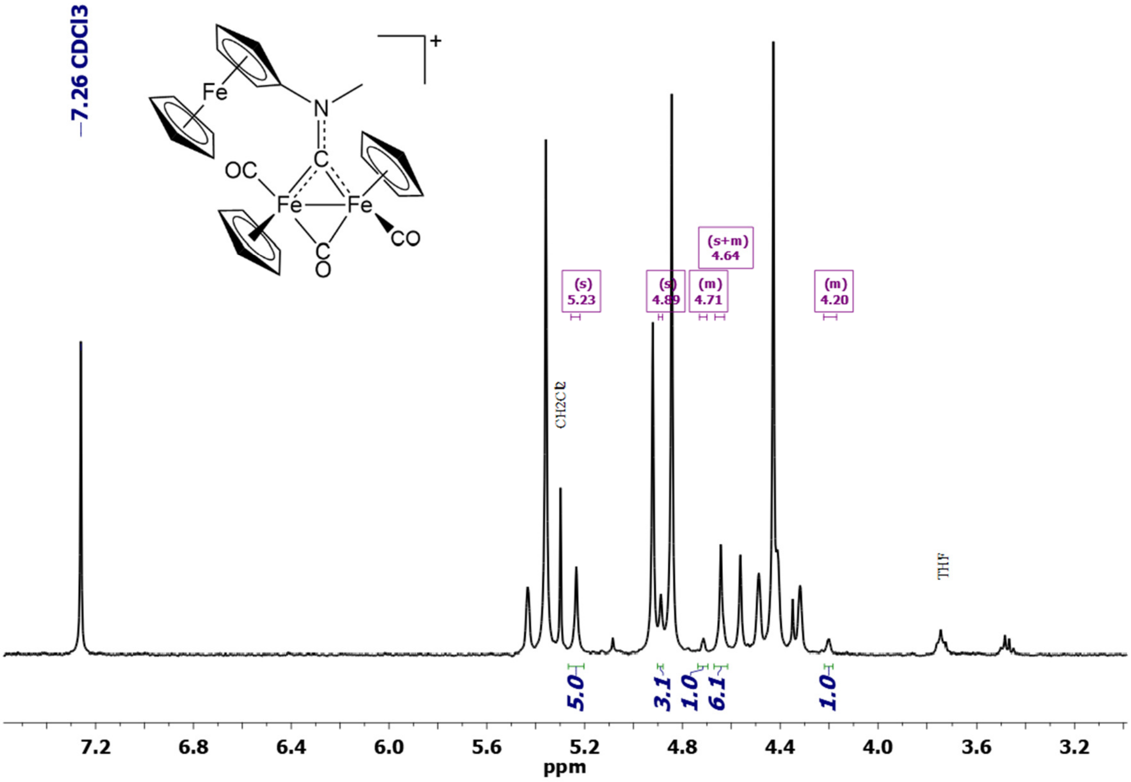

**Figure S8.** $^{13}C\{^{1}H\}$ NMR spectrum (101 MHz, $CDCl_3$) of **[2]CF₃SO₃** (*cis*/*trans* ratio 5:1).

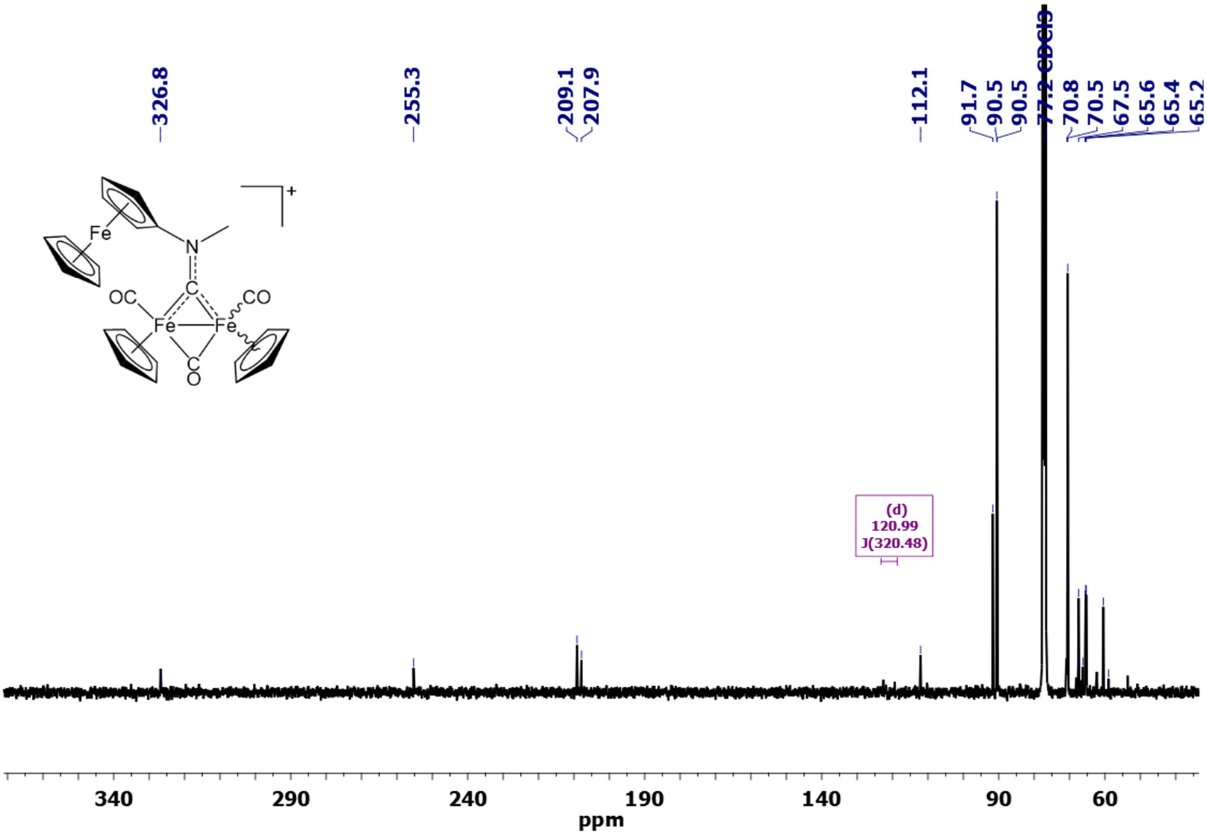


**Figure S9.** Black line: $^{1}H$ NMR spectrum (401 MHz, $CDCl_3$) of **[2]CF₃SO₃** (*cis*/*trans* ratio 5:1). Red line: $^{1}H$ NOESY with irradiation at 5.36 ppm (Cp of the *cis* isomer). Blue line: $^{1}H$ NOESY with irradiation at 5.23 ppm (Cp of the *trans* isomer). Observed NOEs are indicated by the arrows.

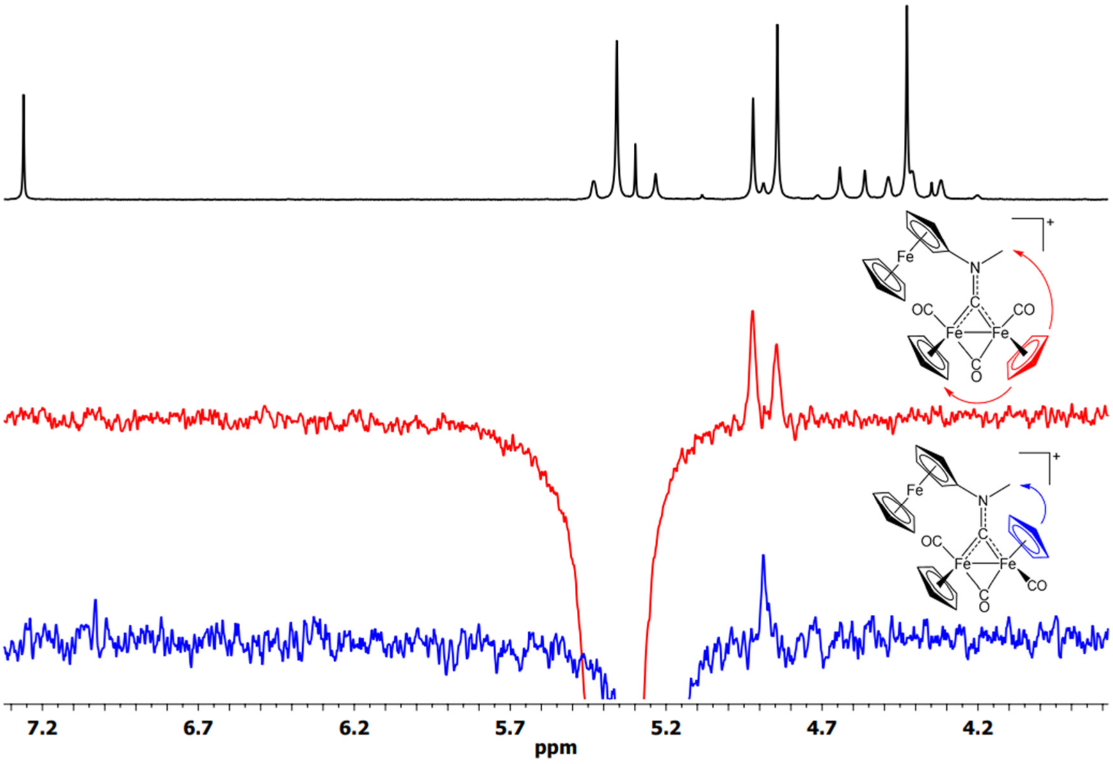

**Figure S10.** IR spectrum (1500-2300 cm$^{-1}$, $CH_2Cl_2$) of [$Fe_2Cp_2(CO)_3(CNPh)$].

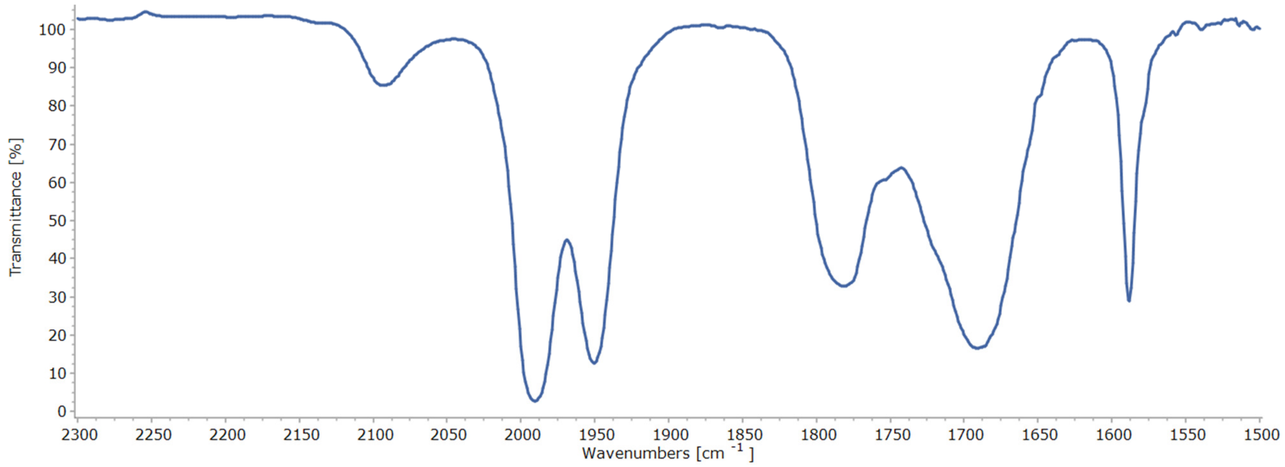


**Figure S11.** IR spectrum (1500-2300 cm$^{-1}$, $CH_2Cl_2$) of [$Fe_2Cp_2(CO)_2$(μ-CO){μ-CN(Me)(Ph)}]$CF_3SO_3$, **[3]$CF_3SO_3$**.

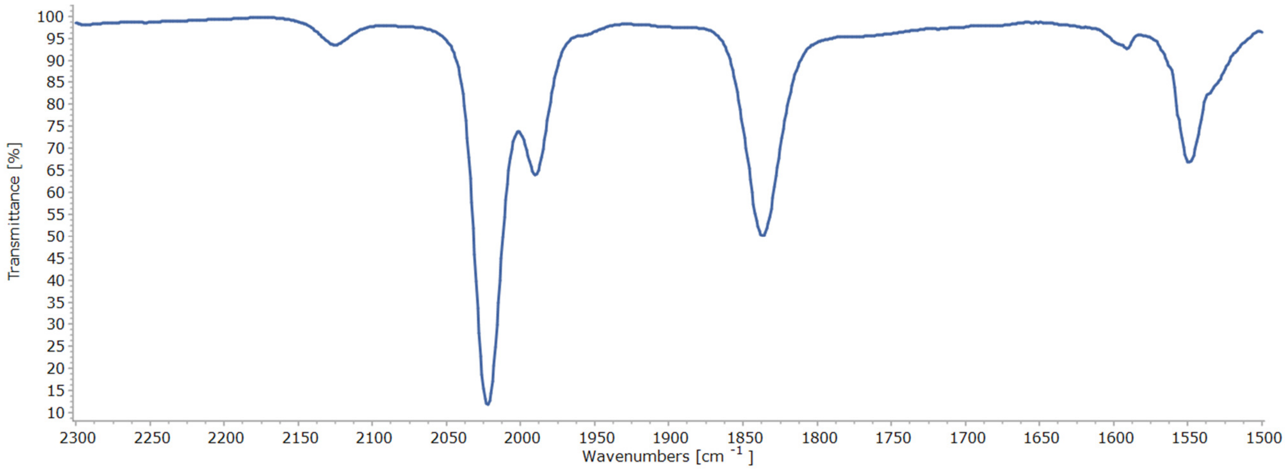

**Figure S12.** $^{1}H$ NMR spectrum (401 MHz, $CDCl_3$) of **[3]CF$_3$SO$_3$**. Signals due to the by-product $[Fe_2Cp_2(CO)(CNPh)(\mu\text{-}CO)\{\mu\text{-}CN(Me)(Ph)\}]^+$ are marked with asterisk (*).

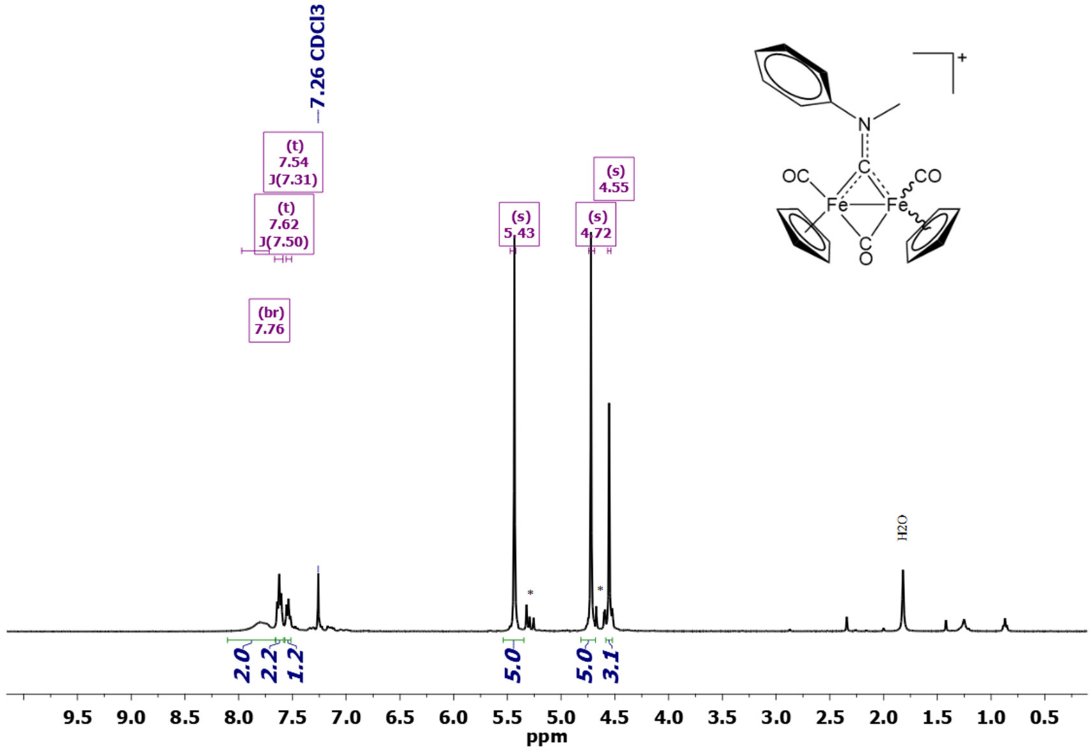

**Figure S13.** $^{13}C\{^{1}H\}$ NMR spectrum (101 MHz, $CDCl_3$) of **[3]CF$_3$SO$_3$**. Signals due to the by-product $[Fe_2Cp_2(CO)(CNPh)(\mu\text{-}CO)\{\mu\text{-}CN(Me)(Ph)\}]^+$ are marked with asterisk (*).

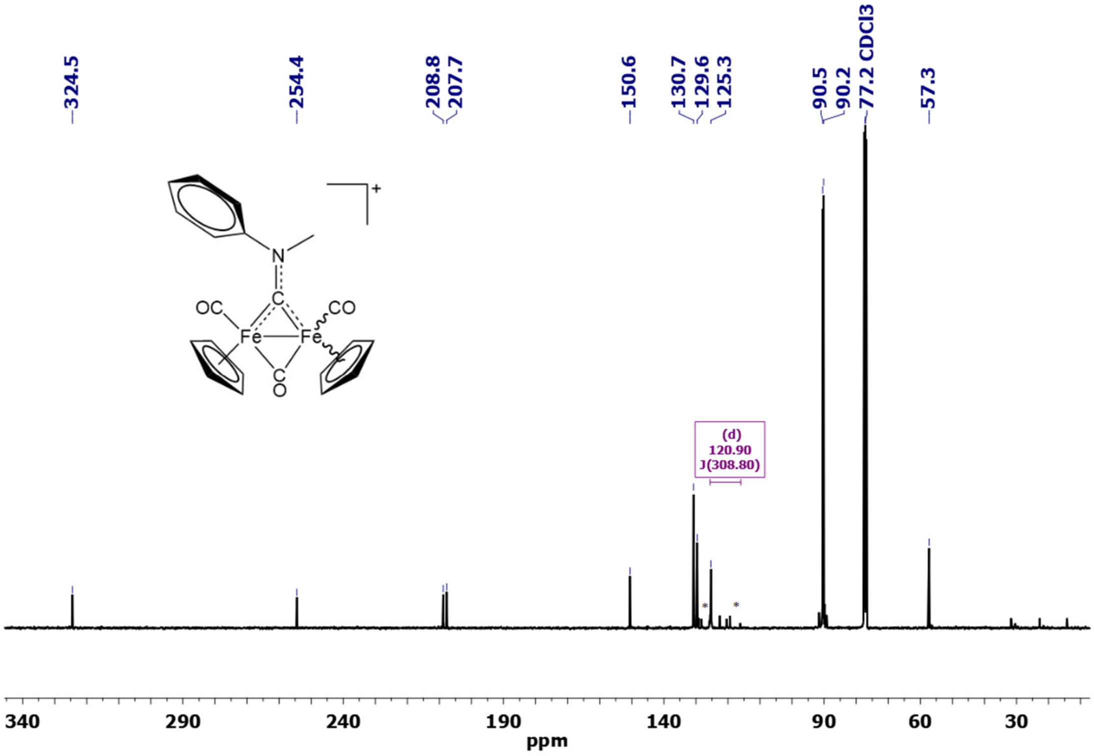

**Figure S14.** Black line: $^{1}$H NMR spectrum (401 MHz, $CDCl_3$) of **[3]$CF_3SO_3$**. Red line: $^{1}$H NOESY with irradiation at 5.43 ppm (Cp). Blue line: $^{1}$H NOESY with irradiation at 4.72 ppm (Cp'). Observed NOEs are indicated by the arrows.

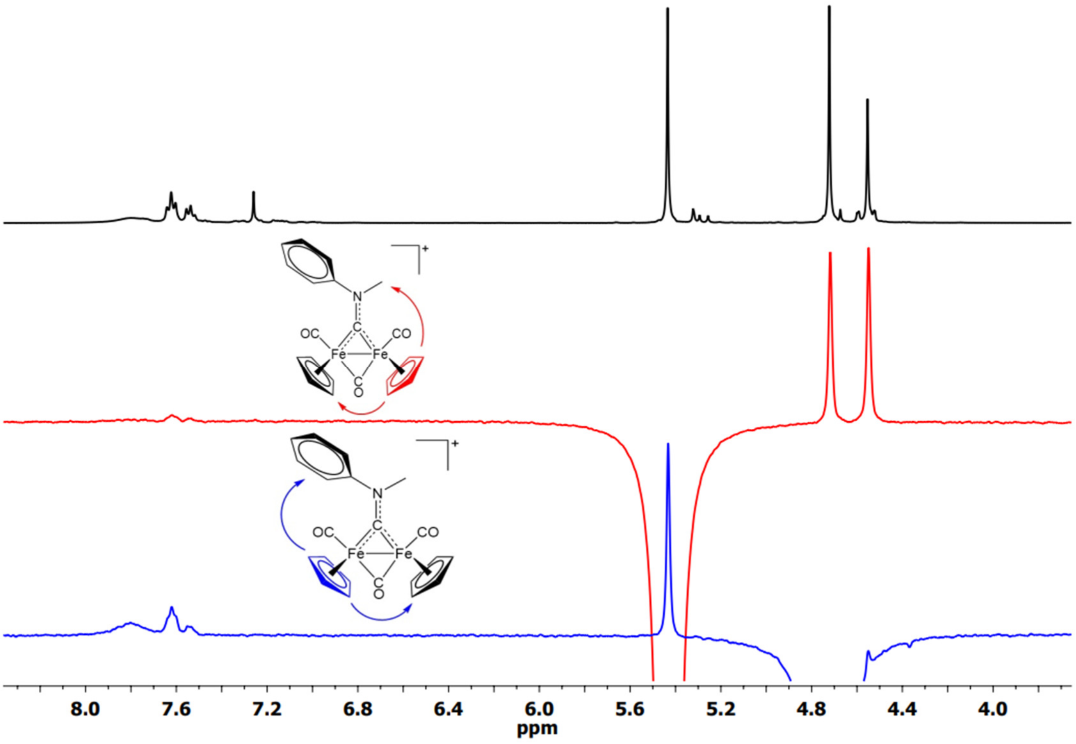

**Figure S15**. DFT optimized geometries of **[3]$^+$**, computed at PBEh-3c level ($CHCl_3$ as continuous medium). Fe, green; N, blue; C, grey. Hydrogen atoms are omitted for clarity.

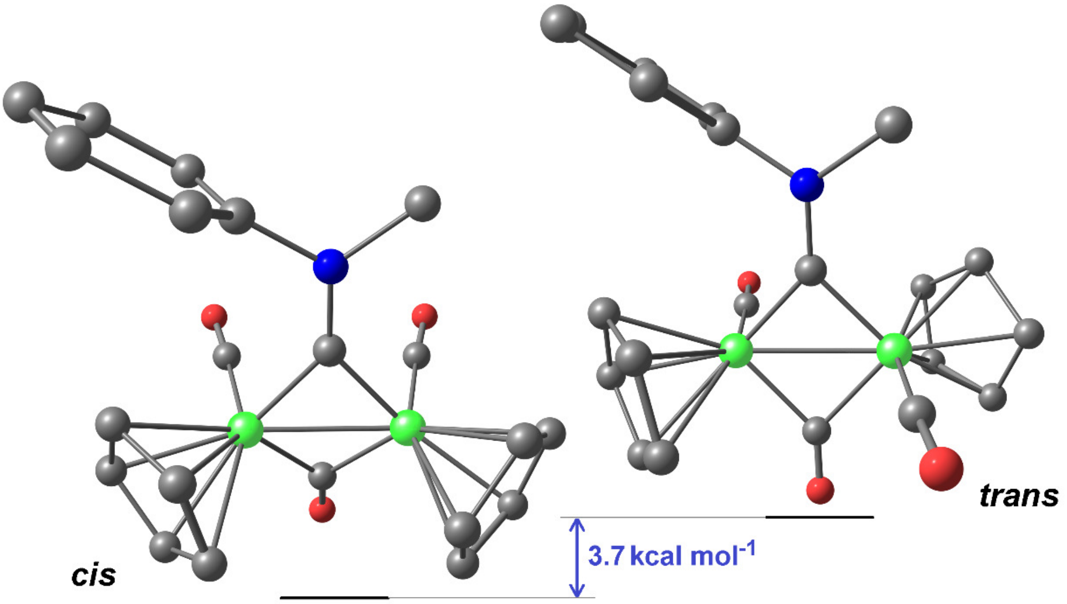

**Figure S16**. Uncorrected simulated IR of **[2]⁺** and **[3]⁺**, computed at PBEh-3c level ($CHCl_3$ as continuous medium). Lorentzian broadening functions, FWHM = 8 $cm^{-1}$.

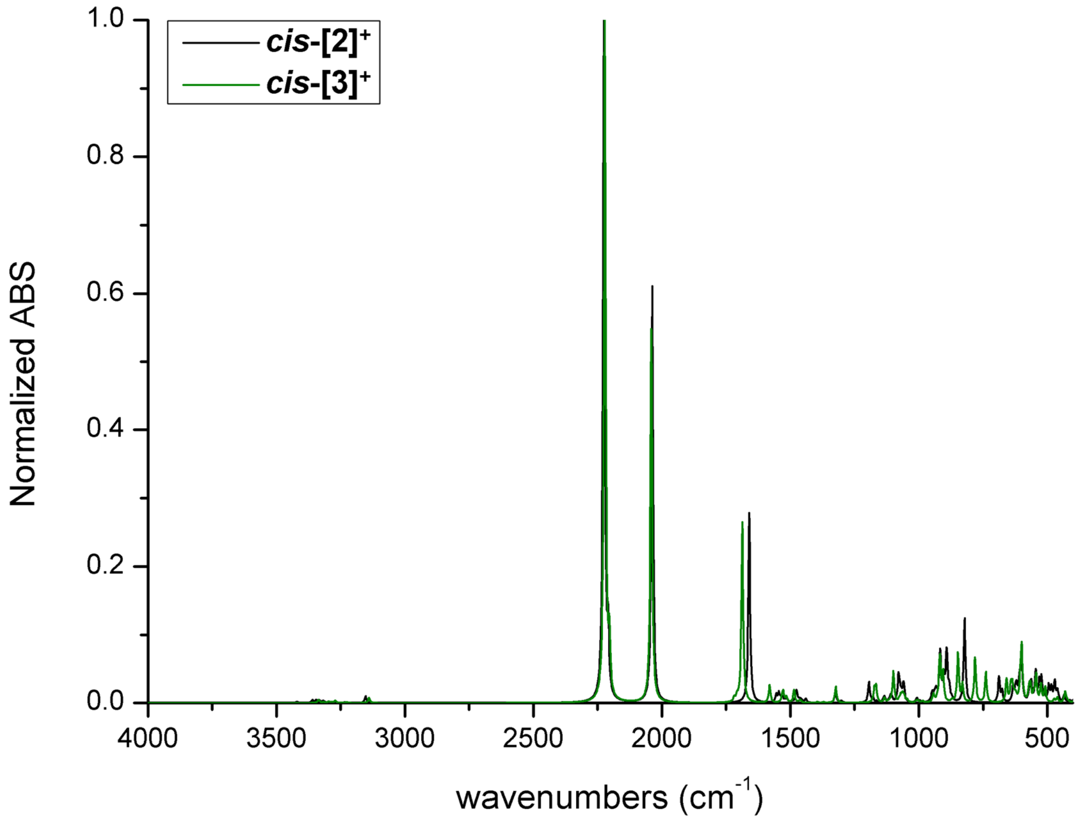


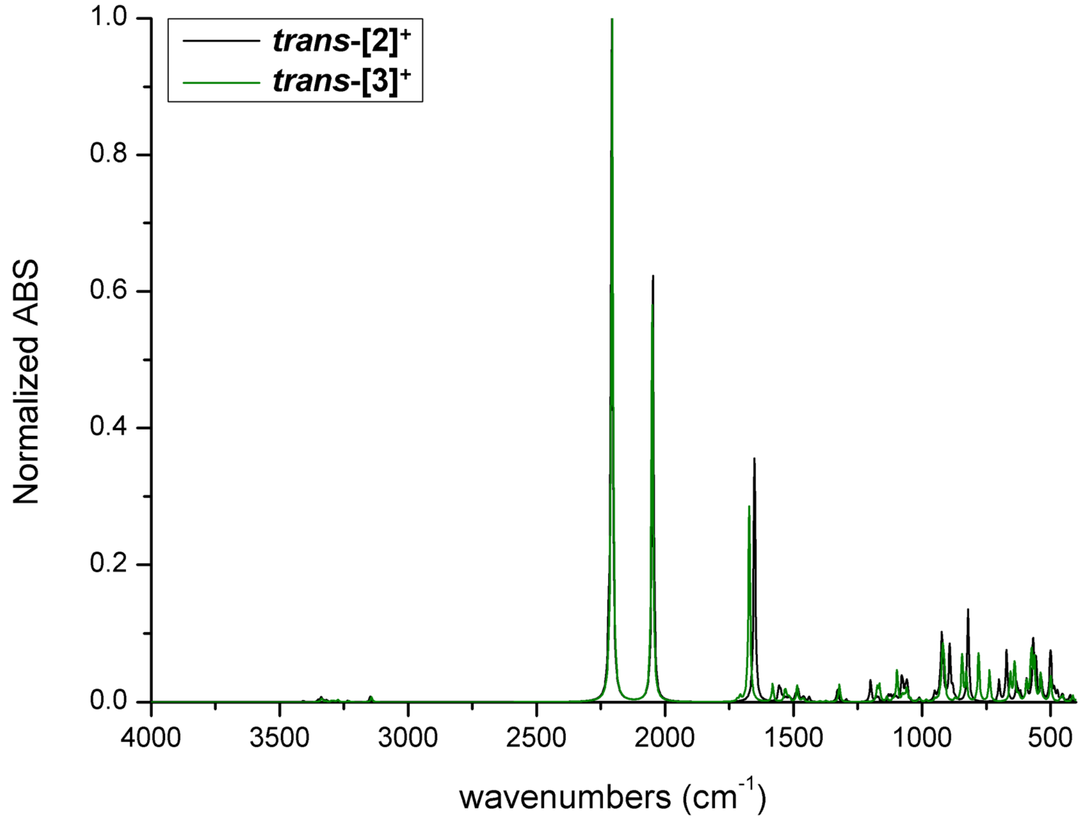

**Figure S17**. IR spectra of a $CH_2Cl_2$ solution of **[2]$CF_3SO_3$** recorded in an OTTLE cell during the progressive decrease of the WE potential a) from –1.3 to –1.6 V; b) from to –1.6 to –1.8 V *(vs* $FeCp_2$; scan rate 1 mV $sec^{-1}$); and c) during a 10 minutes microelectrolysis at the constant potential of –1.8 V. [$N^nBu_4$]$PF_6$ (0.2 mol $dm^{-3}$) as the supporting electrolyte. The absorptions of the solvent and supporting electrolyte have been subtracted.

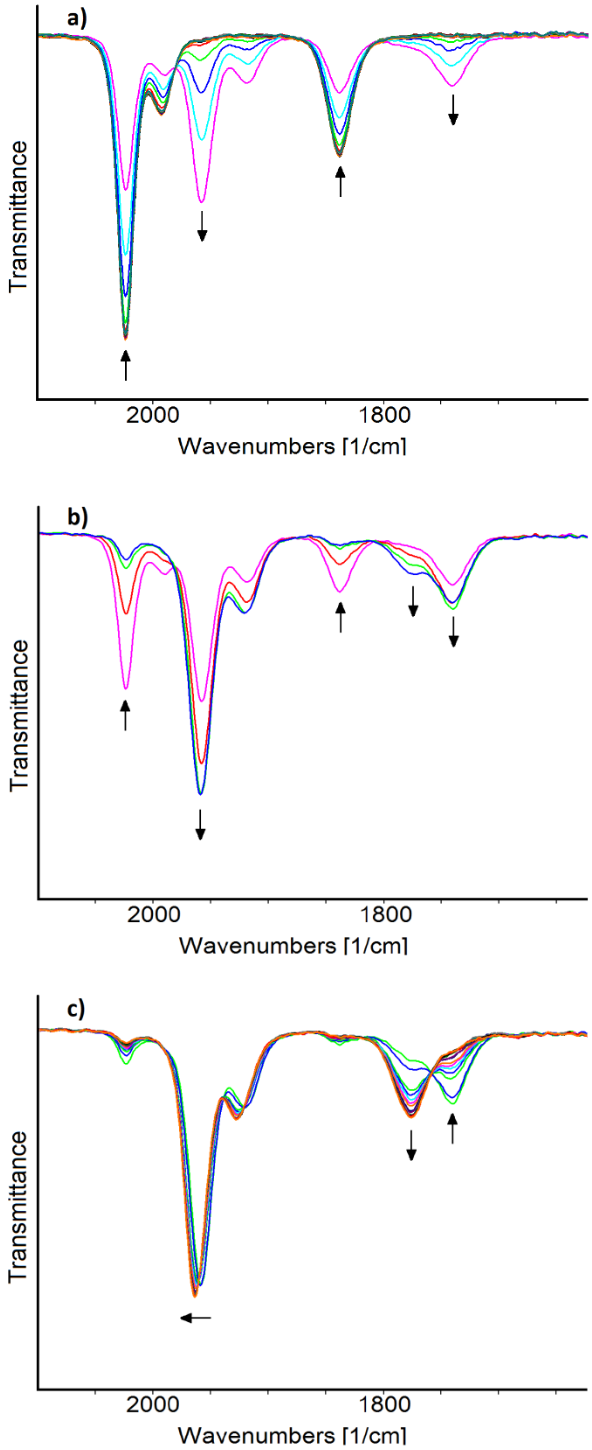

**Figure S18**. IR spectra of a $CH_2Cl_2$ solution of **[2]$CF_3SO_3$** recorded in an OTTLE cell a) during the progressive decrease of the WE potential from –1.3 to –1.8 V (*vs* $FeCp_2$; scan rate 1 mV $sec^{-1}$); b) during 10 minutes in the cell without an applied potential. Starred peak is due to impurities. $[N^nBu_4]PF_6$ (0.2 mol $dm^{-3}$) as the supporting electrolyte. The absorptions of the solvent and supporting electrolyte have been subtracted.

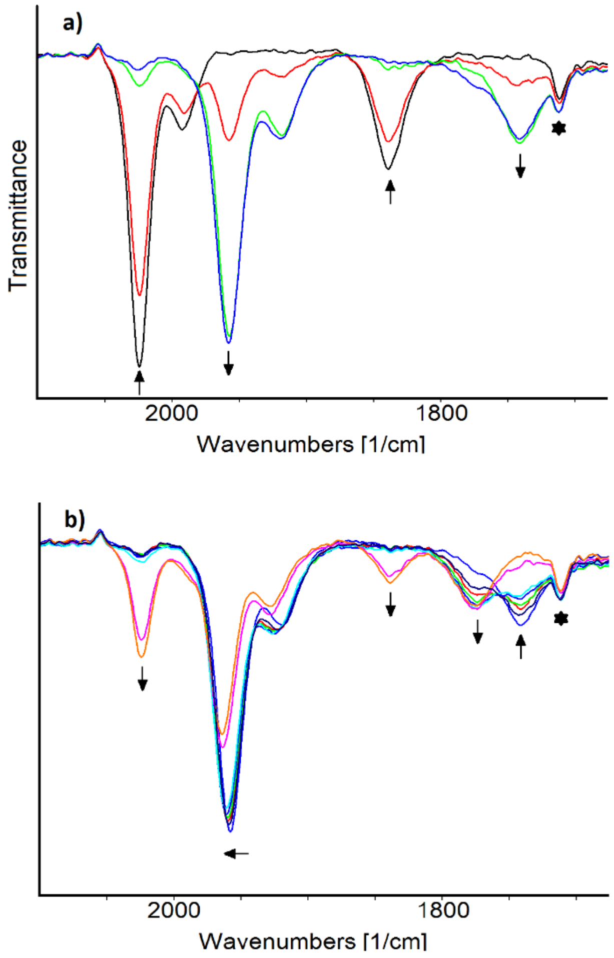


In an effort to determine the electron stoichiometry of the transformation $[\mathbf{2}]^+ \rightarrow \mathbf{2*}$, we subjected a solution of $[\mathbf{2}]^+$ in $CH_2Cl_2$ to constant-potential bulk-electrolysis ($E_w = -1.1$ V, *vs* Ag/AgCl). By IR monitoring of the electrolysis progress, we verified coulometrically that the complete conversion of $[\mathbf{2}]^+$ into the unknown species **2*** requires 2 moles of electrons per mole of complex. The first electron equivalent converts $[\mathbf{2}]^+$ into $[\mathbf{2}]^\bullet$, which slowly consumes a second mole of electrons to quantitatively yield **2*** (Eqs 1 and 2, Figure S19).

$$(1) \quad 2\,[\mathbf{2}]^+ + 2e^- \longrightarrow 2\,[\mathbf{2}]^\bullet$$

$$(2) \quad 2\,[\mathbf{2}]^\bullet \xrightarrow{CH_2Cl_2} [\mathbf{2}]^+ + \mathbf{2}^*$$

**Figure S19**. Profile of the current *vs* time during the bulk electrolysis of a $CH_2Cl_2$ solution of **[2]$CF_3SO_3$** at a Pt electrode. The total charge Q at t = 560 sec corresponds to 1 electron per molecule.

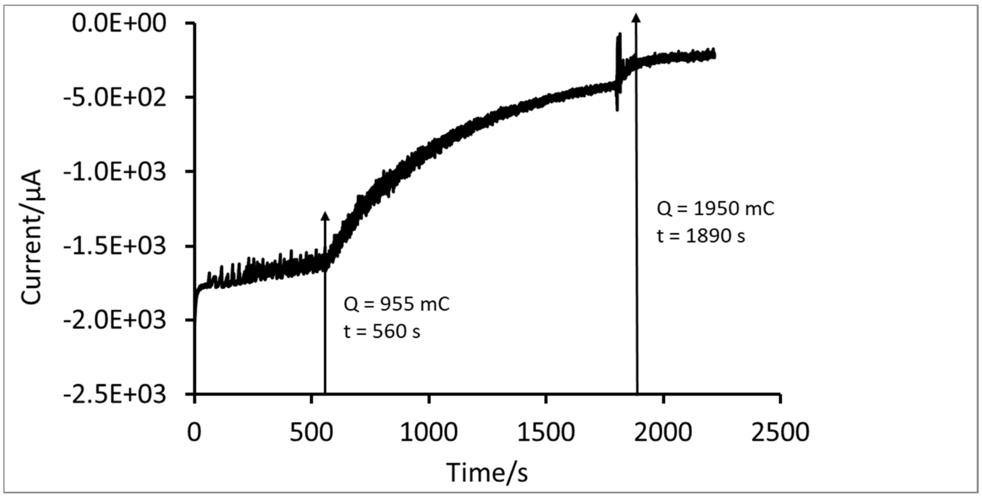


**Figure S20**. IR spectra of a THF solution of **[3]$CF_3SO_3$** recorded in an OTTLE cell during the progressive decrease of the WE potential from –1.2 to –1.6 V (*vs* $FeCp_2$; scan rate 1 mV $sec^{-1}$). Starred peak is due to impurities. $[N^nBu_4]PF_6$ (0.2 mol $dm^{-3}$) as the supporting electrolyte. The absorptions of the solvent and supporting electrolyte have been subtracted.

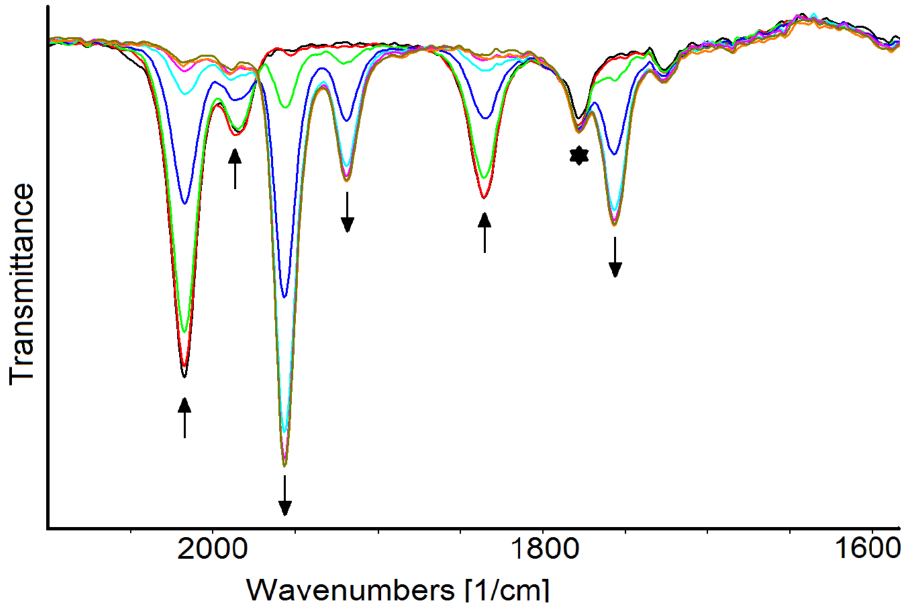

**Figure S21**. CV of **[4]CF$_3$SO$_3$** recorded at a GC electrode in PB (pH = 7.3) solution (scan rate: 0.1 V s$^{-1}$).

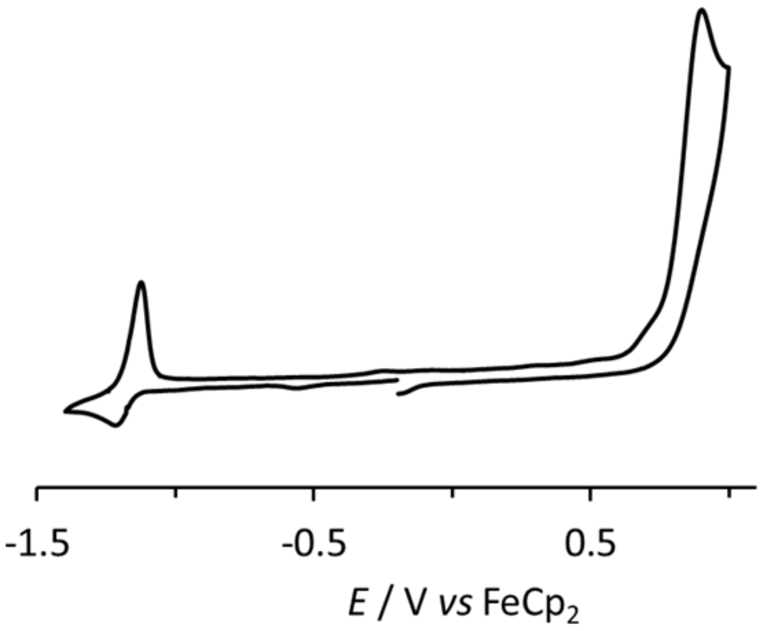

**Figure S22**. Uncorrected simulated IR spectra of **[2]⁺** and **[2]˙**, computed at PBEh-3c level (acetone as continuous medium). Lorentzian broadening functions, FWHM = 8 $cm^{-1}$.

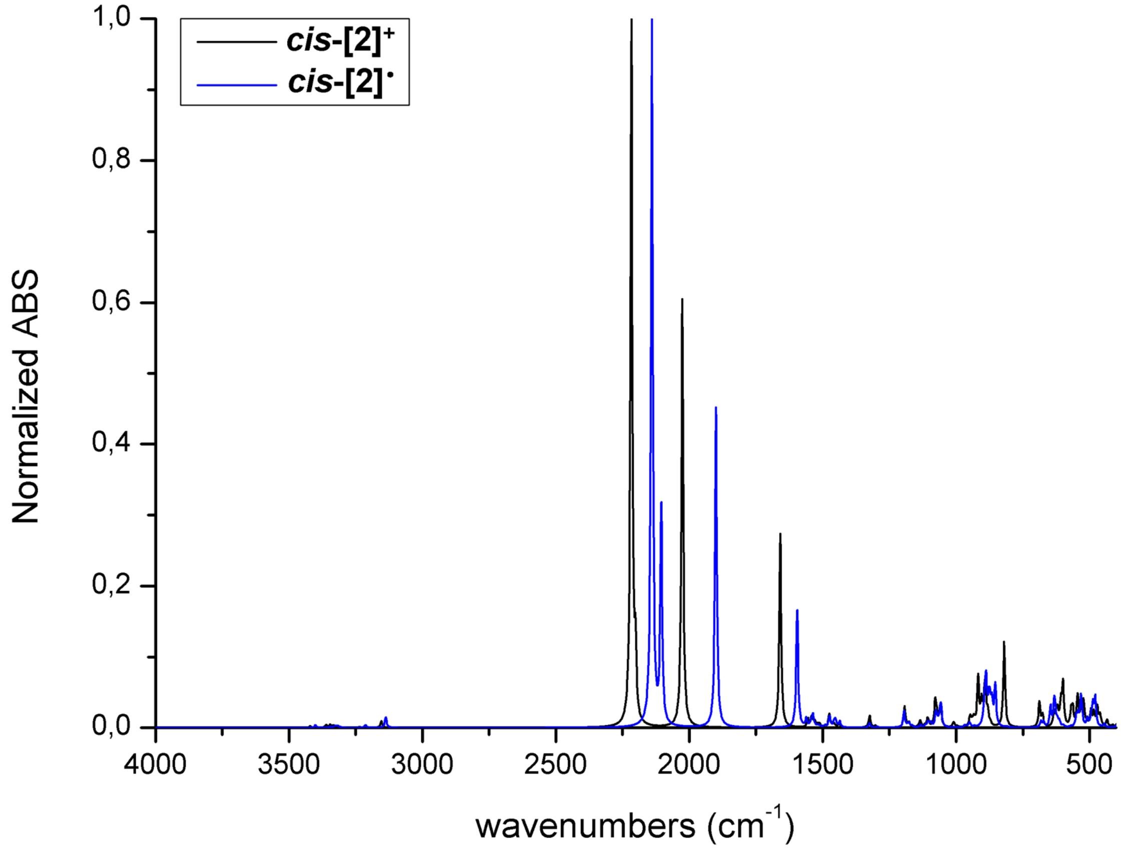


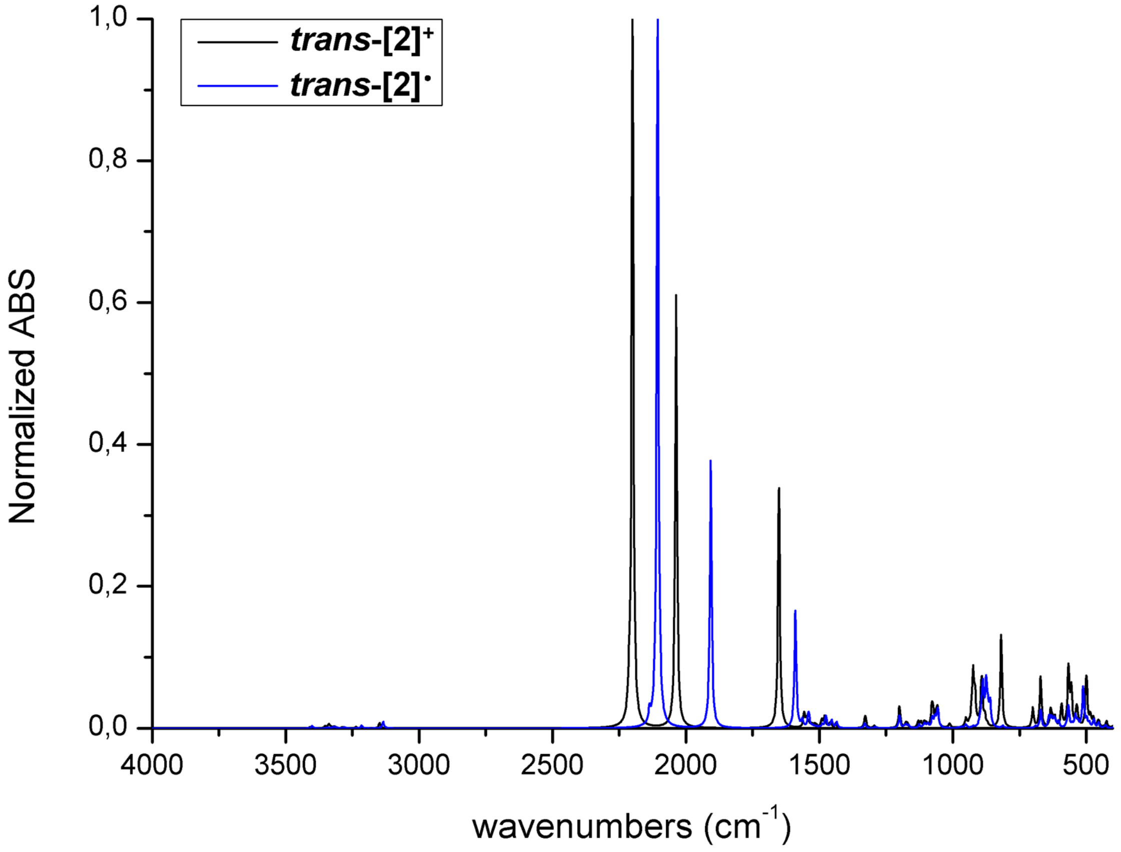

## References


[1] Magdzinski, E.; Gobbo, P.; Martin, C. D.; Workentin, M. S.; Ragogna, P. J. The Syntheses and Electrochemical Studies of a Ferrocene Substituted Diiminopyridine Ligand and Its P, S, Se, and Te Complexes, *Inorg. Chem.* **2012**, *51*, 8425-8432.

[2] Srinivas, P.; Prabhakar, S.; Chevallier, F.; Nassar, E.; Erb, W.; Dorcet, V.; Jouikov, V.; Krishna, P. R.; Mongin, F. Synthesis of ferrocene amides and ester from aminoferrocene and 2-substituited ferocenecorboxylic acid and properties thereof. *New J. Chem.* **2016**, *40*, 9441-9447.

[3] Nieto, D.; Bruna, S.; Gonzalez-Vadillo, A. M.; Perles, J.; Carrillo-Hermosilla, F.; Antinolo, A.; Padron, J. M.; Plata, G. B.; Cuadrado, I. Catalytically Generated Ferrocene-Containing Guanidines as Efficient Precursors for New Redox-Active Heterometallic Platinum(II) Complexes with Anticancer Activity, *Organometallics* **2015**, *34*, 5407−5417.

[4] Goeltz, J. C.; Kubiak, C. P. Facile purification of Iodoferrocene. *Organometallics* **2011**, *30*, 3908-3910.

[5] Dhake, K. P.; Tambade, P. J.; Singhal, R. S.; Bhanage, B. M. An efficient, catalyst- and solvent-free N-formylation of aromatic and aliphatic amines, *Green Chem. Lett. Rev.* **2011**, *4*, 151-157.
